# Inter-Annual Variation in Characteristics of Endozoochory by Wild Japanese Macaques

**DOI:** 10.1371/journal.pone.0108155

**Published:** 2014-10-01

**Authors:** Yamato Tsuji

**Affiliations:** Primate Research Institute, Kyoto University, Inuyama, Aichi, Japan; Institut Pluridisciplinaire Hubert Curien, France

## Abstract

Endozoochory is important to the dynamics and regeneration of forest ecosystems. Despite the universality of inter-annual variation in fruit production, few studies have addressed the sign (seed predation versus seed dispersal) and strength (frequency and quantity) of fruit-frugivore interaction and the effectiveness of endozoochory in response to the long-term temporal context. In this study I evaluated the characteristics of endozoochorous dispersal by wild Japanese macaques *Macaca fuscata* inhabiting deciduous forest in northern Japan for five different years. I collected 378 fecal samples from the macaques in fall (September to November) and quantified the proportion of feces containing seeds, number of seeds per fecal sample, ratio of intact seeds, and seed diversity. The proportion of feces containing seeds of any species (five-year mean: 85.9%, range: 78–97%) did not show significant inter-annual variation, while species-level proportions did. The intact ratio of seeds (mean: 83%, range: 61–98%) varied significantly both between years and between months, and this varied among dominant plant species. The number of seeds per fecal sample (mean: 78, range: 32–102) varied monthly but did not between years, and the seed diversity (mean: 0.66, range: 0.57–0.81) did not show significant inter-annual variation, both of which were attributed to longer duration of macaques’ gastro-intestinal passage time of seeds exceed their feeding bouts. This study demonstrated that frequency and success of seed dispersal over seed predation of macaque endozoochory showed inter-annual variation, indicating low specificity across the seed–macaque network. The temporal variability in the quality of seed dispersal may provide evidence of high resilience in response to fluctuating environmental conditions in the temperate forests.

## Introduction

The context-dependence of species interactions is a key area of ecological studies [1]–[2]. At finer level interactions between a pair of species, temporal variations in the biotic and abiotic conditions can lead to the context-dependence of the strength and sign (i.e., its position along the mutualism-antagonism continuum) of the interactions [2]–[3]. To address the ecological and/or evolutionary meaning of the interactions, it makes sense to document these context-specific phenomena rather than to consider the average outcome over time [3].

Fruits, including fleshy fruits and nuts, are consumed by a wide assemblage of frugivores, especially mammals and birds [4]–[6], which are the principal seed dispersers of fleshy-fruited plant species in forest ecosystems [7]. In general, fruit production is not stable over time, but fluctuates not only seasonally but also inter-annually [8]–[12]. Large temporal changes in fruiting often cause corresponding long-term variation in the diet of frugivorous animals [13]–[16]. This in turn can affect the quantity and quality of seed dispersal, and hence the overall dispersal effectiveness over time [17]. However, despite worldwide research efforts on fruit–frugivore interactions [18]–[19], there have been few studies which addressed the variation in the sign (in terms of seed predation versus seed dispersal) and the strength (frequency and quantity) of fruit–frugivore interactions at the local level [17] but see [2]. To accurately evaluate the fruit–frugivore interaction and the effectiveness of endozoochory, studies covering several years with differing food environment conditions are necessary.

In this study I evaluated the relative importance of inter-annual variations in the sign and strength of fruit–frugivore interactions between fleshy-fruited plants and wild Japanese macaques, *Macaca fuscata* (Cercopithecidae, order Primates). Previous studies of this species have revealed that they disperse seeds of various plant species, especially in fall (September to November) through defecation [20]–[22], and they show inter-annual variation in the degree of fruit-eating [14], [23]. In addition, it has been known that fruiting of many plant species on which the macaques feed (berries) show inter-annual variation [24]–[25]. I, therefore, expected that the macaques would modulate the quantitative and qualitative components of seed dispersal effectiveness via their feeding behavior at different times.

Specifically, I quantified the following four aspects of endozoochory: 1) the proportion of feces containing seeds, 2) the number of seeds per fecal sample, 3) the ratio of intact seeds within a single fecal sample, and 4) the seed species diversity within each single fecal sample in fall for five years (between 2000 and 2008). Based on the inter-annual variation in fruiting of many dietary plants in northern Japan [24]–[25], I predicted that the intact ratio, diversity, frequency of seed occurrence, and number of dispersed seeds of each fruit–macaque pair would not be stable over time and that there would be a low specificity across the fleshy-fruited plants–macaque network in the temperate forest.

## Results

During the study period I collected 388 macaque fecal samples in total, from which I picked up 31,166 seeds from 15 different species (Table 1). I summarized characteristics of each species: life form, fruiting months, size of seeds and fruits, and handling technique by the macaques in Table 2.

**Table 1:** Summary of the data obtained from the faecal samples collected during five study years.

Year/Month	Number of faecal samples	Total number of seeds	*AR* (%)	*SN* (mean±SD)	*IR* (%±SD)	Seed diversity (*H'*±SD)
2000	66	2079	87.9	31.6±58.6	60.6±32.8	0.81±0.30
October	66	2079	87.9	31.6±58.6	60.6±32.8	0.81±0.30
2004	72	7099	83.3	101.6±189.7	87.1±21.3	0.57±0.30
September	3	10	100.0	3.3±3.2	42.9±51.5	0.00±0.00
October	24	2250	87.5	93.9±164.8	90.3±20.1	0.35±0.36
November	45	4839	80.0	108.6±207.0	88.9±14.9	0.47±0.39
2005	57	3720	96.5	65.3±109.0	97.6±9.1	0.66±0.33
September	11	446	81.8	32.6±51.0	90.2±20.2	0.42±0.39
October	31	1302	100.0	42.0±40.6	98.7±4.7	0.47±0.41
November	15	1972	100.0	131.5±191.4	99.9±0.2	0.31±0.36
2007	77	7426	83.1	96.5±132.5	86.4±20.2	0.63±0.32
September	29	1322	89.7	45.6±55.3	86.2±24.7	0.49±0.33
October	25	2424	100.0	97.0±75.3	80.0±17.7	0.75±0.34
November	23	3680	56.5	160.2±209.0	99.3±1.6	0.47±0.31
2008	116	10842	78.4	94.2±123.8	84.4±24.4	0.65±0.33
September	36	1234	50.0	68.6±46.2	78.4±27.6	0.44±0.24
October	41	1359	82.9	33.3±39.2	76.9±27.3	0.78±0.36
November	39	8249	100.0	213.6±145.2	93.8±16.7	0.58±0.33
5- Year Total	388	31166				
Mean			85.9	77.8±122.7	83.2±21.6	0.66±0.32

Seed diversity has been calculated at the Shannon Winer index (*H'*).

*AR*: appearance ration of seeds, *SN*: Number of seeds per faecal sample, *IR*: intact ratio of seeds.

**Table 2:** Seed found within fecal samples of Japanese macaques on Kinkazn, northern Japan (fall in 2000, 2004, 2005, 2007, and 2008).

					Seed size^d^		# Seeds per fruit^d^	Fruit size^e^			
Species	Family	Life from^a^	Fruiting^b^	SHT^c^	mm	mm^3^		mm	mm^3^	Relative volume of seeds (%)^f^	
*Berchemia racemosa*	Rhamnaceae	V	-Sep	S	3.7	26.5	1.0	5.0	65.4	40.5	
*Cornus kousa*	Cornaceae	H	Sep–Oct	D	4.5	47.7	1.0	15.7	2026.3	2.4	
*Schisandra nigra*	Schisandraceae	V	Sep–Oct	S	4.4	44.6	2.0	–	810.5	11.0	
*Tubocapsicum anomalum*	Solanaceae	H	Sep–Nov	S	1.3	1.2	117.8	–	–	–	
*Swida macrophylla*	Cornaceae	HT	Sep–Nov	C	3.4	20.6	2.0	–	97.4	42.3	
*Malus tschonoskii*	Rosaceae	HT	Sep–Nov	S	3.5	22.4	7.5	–	4717.1	3.6	
*Pourthiaea villosa*	Rosaceae	S	Sep–Nov	S	2.7	10.3	3.3	–	325.9	10.4	
*Zanthoxylum piperitum*	Rutaceae	S	Sep–Nov	C	2.8	11.5	1.0	4.3	41.6	27.6	
*Amphicarpaea brachteata*	Leguminosae	H	Sep–Nov	C	1.6	2.1	2.8	–	–	–	
*Vitis flexuosa*	Vitaceae	V	Sep–Nov	S	3.5	22.4	3.3	9.0	381.7	19.4	
*Sorbus japonica*	Rosaceae	H	Sep–Nov	S	3.1	15.6	2.0	9.0	63.9	48.8	
*Rosa multiflora*	Rosaceae	S	Oct–Nov	S	2.2	5.6	7.0	7.5	220.9	17.7	
*Vibrnum dilatatum*	Caprifoliaceae	S	Oct–Nov	S	3.3	18.8	1.0	6.0	113.1	16.6	
*Perilla frutescens*	Labiatae	H	Oct–Nov	C	1.7	2.6	4.0	–	–	–	
*Viscum album*	Loranthaceae	HP	Nov–	S	4.0	33.5	1.0	–	–	–	

Life form, fruiting months, sizes of both seeds and fruits, and seed handling technique by the macaques are also shown.

a H: herbaceous plants, HT: high tree, HP: hemi-parasite, V: vine, S: shrub, obtained from [22].

b Tsuji, unpublished data.

c Seed handling technique (C: crunching, D: discarded, S: swallowing) obtained from [22].

d obtained from [22].

e obtained from [15], [42]–[43].

f calculated by (total seed volume/fruit volume×100).

**Figure 1:**
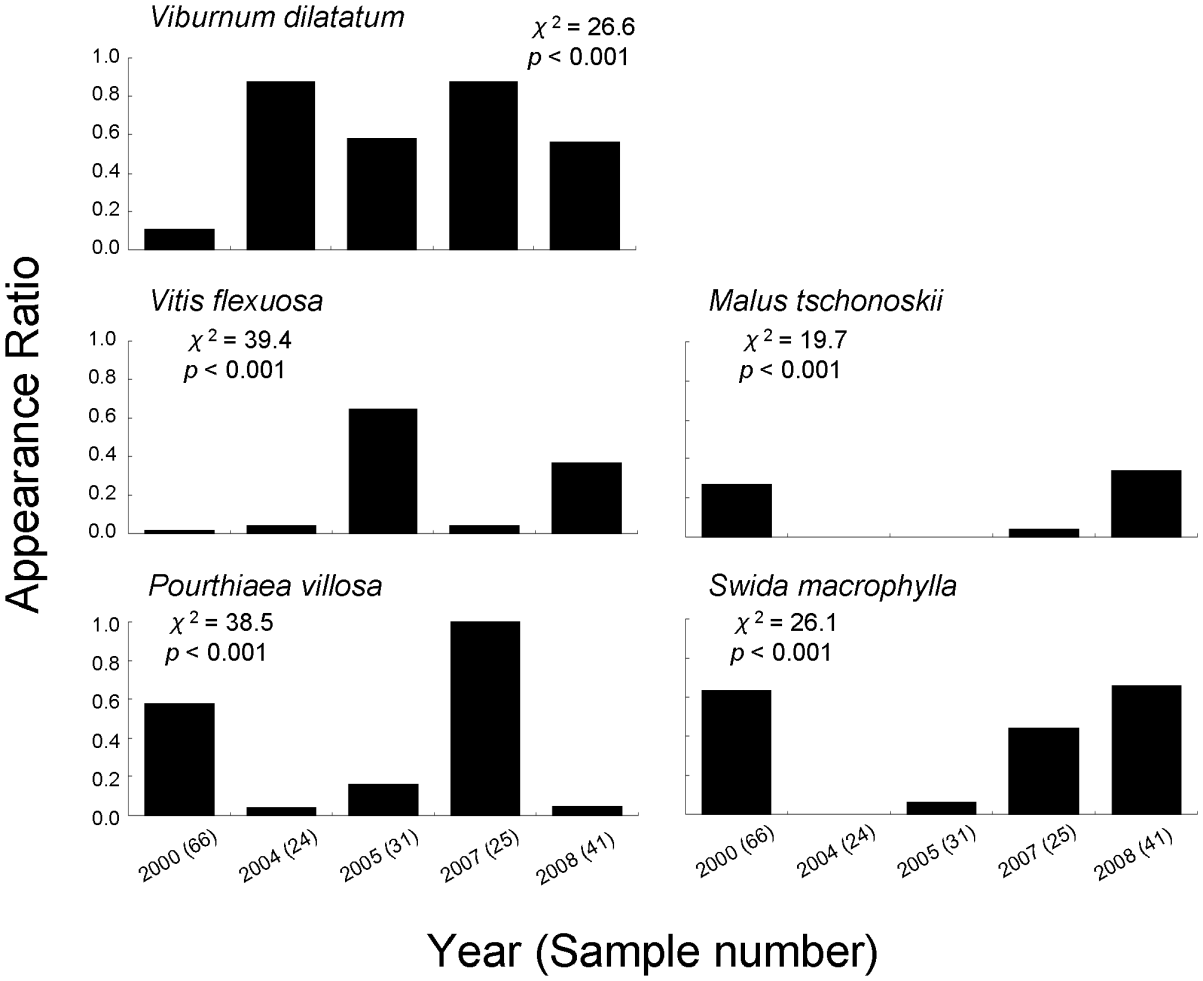
Inter-annual change in the seed appearance ratio of five major plant species from fecal samples collected in October. Samples were collected between 2000 and 2008. Figures in parentheses represent the number of analyzed samples in a given year. Results of statistical analyses (chi-square tests) are also shown.

Seed appearance ratios (*AR*s) for all species combined ranged from 78% (in 2008) to 97% (in 2005), and showed no significant variations among months and among years. An effect of interaction between year and month was not detected (Table 3). At the finer (i.e., species) level, however, I obtained significant inter-annual variations in the *AR*s; for example, in October, the *AR* of *Viburnum dilatatum* (lower in 2000), *Vitis flexuosa* (higher in 2005 and 2008), *Pourthiaea villosa* (higher in 2000 and 2007), *Malus tschonoskii* (higher in 2000 and 2008), and *Swida macrophylla* (lower in 2004 and 2005) showed significant inter-annual variations in the *AR*s (Fig. 1).

**Table 3:** Factors affecting seed apparent ratio (AR), seed number per fecal sample (SN), intact ratio of seeds (IR), and seed diversity (H') revealed by the GLM analyses using month, year, dominant plant species (*DPS*), and their interactions as independent variables.

Variables	Main effects						Interactions							
	Month		Year		*DPS*		Month × Year		Month × *DPS*		Year × *DPS*		Month × Year × *DPS*	
Apparent ratio (*AR*)	*z* = –1.263	*p* = 0.207	*z* = 1.393	*p* = 0.371	–	–	*z* = 0.930	*p* = 0.353	–	–	–	–	–	–
Number of seeds (*SN*)	*z* = 1.978	*p = *0.048*	*z* = 0.899	*p* = 0.369	*z* = 1.203	*p* = 0.229	*z* = –0.778	*p* = 0.436	*z* = –1.299	*p* = 0.194	*z* = –0.621	*p* = 0.535	*z* = 0.649	*p* = 0.516
Intact ratio *(IR*)	*z* = 2.365	*p = *0.018*	*z* = 2.531	*p* = 0.011*	*z* = 2.073	*p* = 0.038*	*z* = –2.362	*p* = 0.018*	*z* = –2.006	*p* = 0.045*	*z* = –2.072	*p* = 0.038*	*z* = 2.005	*p* = 0.045*
Seed diversity (*H'*)	*t* = 0.840	*p* = 0.402	*t* = 0.209	*p* = 0.835	*t* = 0.367	*p* = 0.714	*t* = 1.539	*p* = 0.134	*t* = 0.663	*p* = 0.512	*t* = 0.491	*p* = 0.627	*t* = –0.725	*p* = 0.474

*DPS*: dominant plant species (see methods in text), *: *p*<0.05.

**Figure 2:**
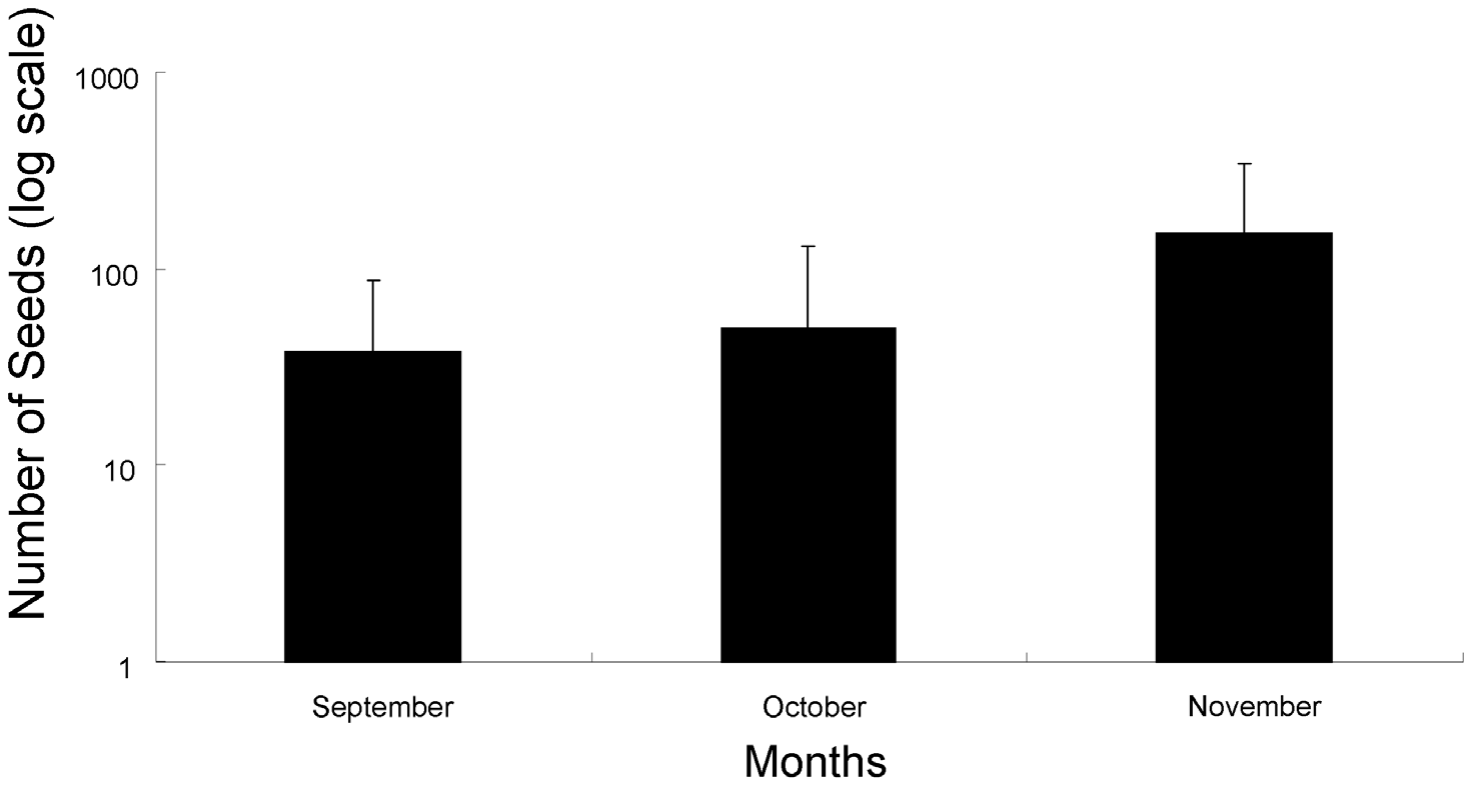
Monthly change in mean (±SD) number of seeds per fecal sample of the Japanese macaques on Kinkazan Island, northern Japan. Samples were collected between 2000 and 2008.

The mean number of seeds per single fecal sample (*SN*) ranged from 32 (in 2000) to 102 (in 2004) (Table 1). Mean (± SD) *SN* differed monthly (Table 3), and was the greatest in November (153±190), middle in October (50±81), and the smallest in September (38±50) (Fig. 2). Effects of the year, the dominant plant species (*DPS*), and all interactions (year × month, year × *DPS*, month × *DPS*, year × month × *DPS*) on the *SN* were not significant (Table 3).

**Figure 3:**
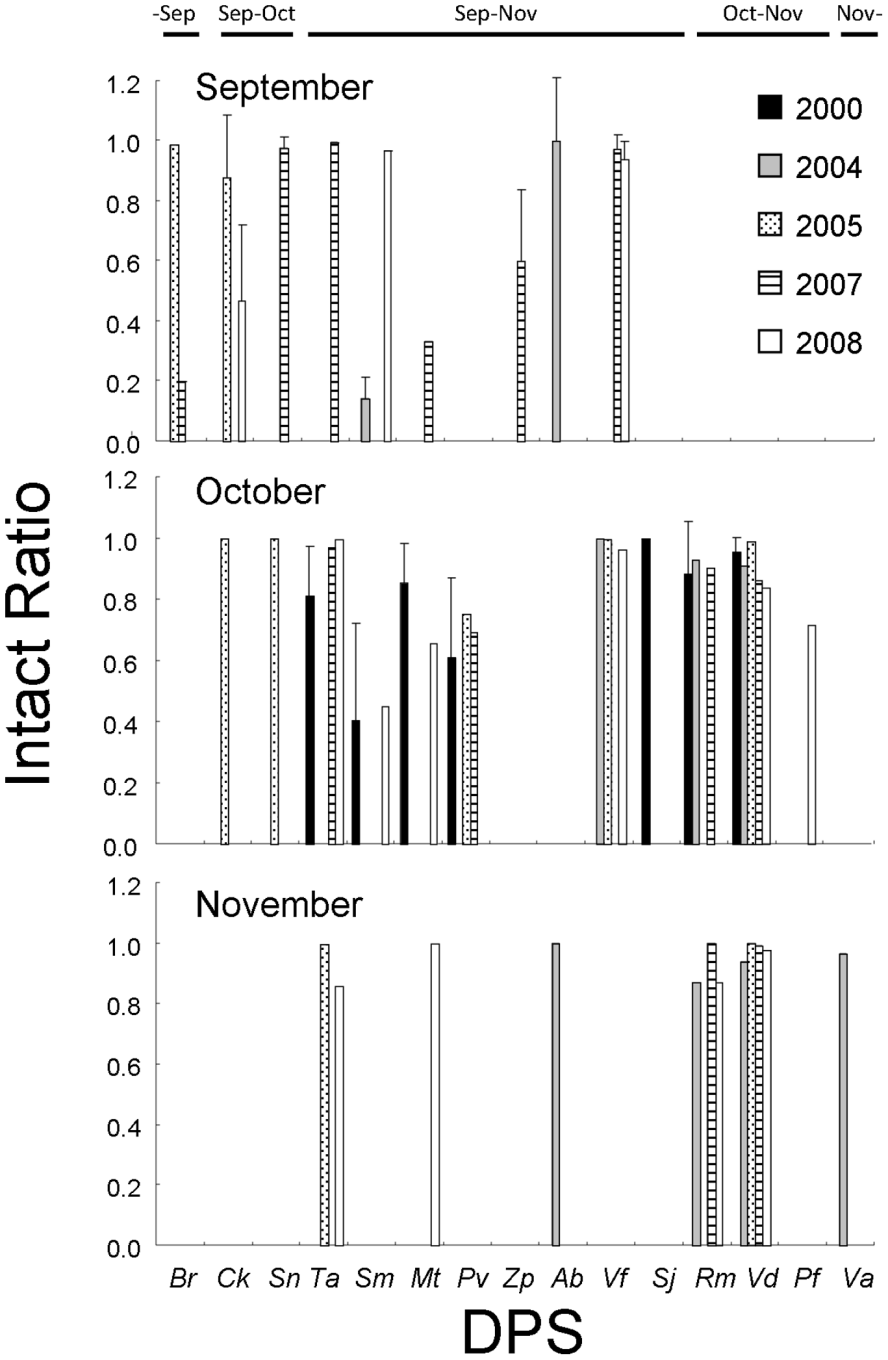
Inter-annual change in mean (±SD) intact ratio of seeds of the Japanese macaques for the dominant plant species in September (top), October (middle), and November (bottom). The intact ratio is obtained by dividing total number of intact seeds by total number of seeds in a given sample. Fruiting months of each species are shown on the top of the figure. Date from 2000 is only from October. *Br* = *Berchemia racemosa*, *Ck* = *Cornus kousa*, *Sn* = *Schisandra nigra*, *Ta* = *Tubocapsicum anomalum*, *Sm* = *Swida macrophylla*, *Mt* = *Malus tschonoskii*, *Pv* = *Pourthiaea villosa*, *Zp* = *Zanthoxylum piperitum*, *Ab* = *Amphicarpaea brachteata*, *Vf* = *Vitis flexuosa*, *Sj* = *Sorbus japonica*, *Rm* = *Rosa multiflora*, *Vd* = *Viburnum dilatatum*, *Pf* = *Perilla frutescens*, *Va* = *Viscum album*.

The seed intact ratio (*IR*) ranged from 61% (in 2000) to 98% (in 2005) (Table 1). Effects of year, month, *DPS*, and, and all interactions on the *IR* were also significant (Table 3); that is, the effect of the *DPS* on the *IR* varied inter-annually and monthly. For example, the *IR*s of seeds composed mainly of *Swida macrophylla* and *Malus tschonoskii* were lower in 2004 and in 2007, respectively, while the *IR*s of seeds composed mainly of *Viburnum dilatatum* and *Rosa multiflora* were stable throughout the study period. Further, the degree of inter-annual variation in the *IR* was larger in September: *Berchemia racemosa*, *Cornus kousa*, and *Swida macrophylla* were typical examples (Fig. 3).

Seed diversity (*H′*) ranged from 0.57 (in 2004) to 0.81 (in 2000). The effects of year, month, *DPS*, and all interactions on the *H′* were not significant (Table 3).

## Discussion

Among the four aspects of endozoochory I examined, the intact ratio of seeds (*IR*) changed significantly among years and among major plant species, and the degree of them varied among dominant plants. Further, the proportion of feces containing seeds (*AR*) at the species level also changed among years. These points imply that the quality and quantity of the endozoochory performed by wild Japanese macaques are not species-specific, but vary with the temporal (i.e., monthly and inter-annually) context, as pointed out for other animals [2], [17].

The cause for the inter-annual variation in the species-level *AR* was elucidated. The most likely factor affecting this is inter-annual variation in fruit production, though I do not have data supporting this. In general, Japanese macaques feed on various kinds of fleshy-fruited plants, probably due to their opportunistic feeding nature [26]–[27]. Furthermore, the macaques flexibly shift fruit species consumption in response to fruit availability [14], [23]. Many fleshy fruited plants in my study site are known to show inter-annual variation in fruiting [24]–[25]; in the years when some fleshy fruits are scarce, they relied on alternative food sources such as unripe fruits or seeds, or the subset of plants bearing fruits during that year *sensu* [9]. Such temporal context-dependence would lead to a lack of specificity in the plant–macaque network, which could enhance its resilience to fluctuating environmental conditions in the temperate region. From this viewpoint, fleshy-fruited plants in the temperate forest are likely to lessen the risk of a completely inappropriate seed treatment by the macaques, and is likely to increase the probability of successful dispersal whatever conditions prevail, as observed in Mediterranean ecosystems [2].

*IR* also changed among years. This was similar to the case of *Cercopithecus* monkeys (guenons) inhabiting tropical Africa, which altered their seed handling technique (swallowing or masticating) according to fruit availability, and *IR* decreased in the year when fruits were scarce and the guenons performed mastication of the seeds within fruits [9]. In the temperate region, seasonality in fruiting is quite clear [28], and the macaques can find some fruits in the fruiting season (fall). Thus, it is not likely that inter-annual changes in the fruit-handling technique by the macaques occur. Rather, inter-annual changes in the *IR* of seeds would be attributed to the type of available fruits at any time. It is known that the size of seeds eaten by macaques has a negative effect on the *IR* [22]; the *IR* of seeds composed mainly of large-sized species is lower than that of small-sized seeds. Thus, in years when only fruit species with relatively large seeds (>3 mm, for example) were available, *IR* would become lower. This tendency might be strong in September in which number of potentially consumed fruits is smaller (11 species over 8 year study) than October and November (20 species for each month, [22]). My result supported this idea.

Unlike the above two characteristics, the number of seeds per fecal sample (*SN*) and seed diversity (*H′*) per fecal sample was consistent throughout the study period (though the former variable changed monthly). It is known that the gastrointestinal passage time of captive Japanese macaques is 37–54 h [29]–[30], which is far longer than the feeding bout length in a fruit patch (ca. 5–25 min) [31]–[32], and single fecal samples contain seeds of multiple plant species ingested across several feeding bouts. Also, the number of seeds within a single fruit (<10 seeds, except for *Tubocapsicum anomalum* having >100 seeds per fruit), does not vary among 15 species investigated here (Table 2). Therefore, it is unlikely that feeding on specific fruits affects the density of seeds within single feces/at defecation sites, and consequent competition among seedlings over resources.

Mutualisms are typically characterized by costs and benefits being highly dependent on the community context to which they belong [3]. The Japanese macaques have broad diets, and are not closely dependent on the limited number of fleshy-fruited plants in any season [26]–[27]. Thus, the ecological variability and conditional outcomes of plant–macaque interactions should be treated as less-extreme fitness costs in less-obligate interactions *sensu* [3]. For several plants, specific dispersal patterns performed by the macaques in extreme years can have a more significant implication than the patterns of typical years. Therefore, evaluating the performance of the dispersers over a longer time scale is important. This possibility can be tested by estimating seed rain and evaluating germination and growth across multiple years when the fruiting and related feeding behavior of dispersers differ. From this viewpoint, long-term monitoring of fruiting of major diet fruits should be conducted.

This study revealed that variations in the frequency and success of seed dispersal over seed predation are affected by the temporal context, while abundance and diversity of seeds are not. My conclusion supports the generally accepted idea of the context-dependence of species interactions [1], [3]. The finding that the context-dependence of endozoochorous dispersal does occur over time seems to be a highly generalized process at other sites in the forest ecosystems. Studies of primates and other frugivorous mammals in temperate regions to date have shown that home range size and its utilization (e.g., [23], [33]–[35]) changes inter-annually. By combining the results of those studies and the present one, researchers can draw conclusions about patterns of seed dispersal, such as the distance from mother trees and the degree of seed concentration in different years (e.g., [36]).

## Materials and Methods

### Ethics statement

I did not need to specific permission for conducting behavioral observation of the macaques and fecal sample collection on Kinkazan Island because this island is considered as public property. My field study did not involve endangered or protected animal/plant species. The research methodology complied with protocols approved by the guidelines (Guide for the Care and Use of Laboratory Primates, Second Edition) of Primate Research Institute, Kyoto University, Japan and the legal requirements of Japan.

### Study site and subjects

Kinkazan Island (38.3 N, 141.6 E) is located 700 m from the Oshika Peninsula, northern Japan. The total area of the island is ca. 9.6 km^2^, and the highest peak is 450 m a.s.l. The monthly mean air temperature on the island ranges from 2.5°C in February to 22.3°C in August. Based on the climate conditions, the year was divided into four seasons: spring (March–May), summer (June–August), fall (September–November), and winter (December–February) [22]. Deciduous forests of *Fagus crenata* (Fagaceae) dominate the higher elevations (>150 m), whereas a mixture of deciduous forests of *Carpinus* spp. (both *C. tschonoskii* and *C. laxiflora*, Betulaceae) and coniferous forests of *Abies firma* (Pinaceae) cover the lower elevations (<150 m) on the island. Approximately 200–250 Japanese macaques belonging to six troops (A, B_1_, B_2_, C_1_, C_2_, and D) inhabit the island [33].

### Fecal sample collection and analysis

I collected fresh fecal samples from adult females in the fall in 2000, 2004, 2005, 2007, and 2008 during the behavioral observation of troop A (16 surveys in total, 2000: October, 2004–2008: September, October, and November), details of the behavioral observation are shown in my previous studies [37]–[38]. I was in contact with the macaques (ca. 10 m in distance) during my observation. Each fecal sample was thoroughly mixed with water and rinsed through 0.5 mm sieves under fresh water. I then picked up all seeds from the fecal samples, identified the seeds to the species level, and counted the seeds of each species. Seed identification was based on my reference collection that had been prepared previously [22], [39].

I evaluated the seed characteristics based on previous studies [22], [40]. I first calculated the number of dispersal events, which was defined as the number of fecal samples containing seeds, for each year for each plant species. I also parsed data by month because fruit vary in abundance during specific weeks and three months window seemed broad. Since I did not collect fecal samples in equal quantities across the five years (range: 57–116), I calculated seed appearance ratios (*AR* hereafter), obtained by dividing the dispersal events by the number of fecal samples examined for each year for each target species. I calculated the *AR* both for all species combined and for each species separately. For the latter analyses I used fecal samples collected in October to eliminate the difference in collecting effort among fall months in each year. For each fecal sample from which seeds appeared, I counted the number of seeds per fecal sample (*SN*). I further calculated the seed intact ratio (*IR*), that is, the ratio of seeds with no apparent physical damage after gut passage, as a qualitative index of the efficacy of seed dispersal. The *IR* was obtained by dividing the total number of intact seeds by the total number of seeds in a given sample. Finally, as an index of seed diversity, I calculated the Shannon-Wiener Index (*H′*) for each fecal sample containing seeds as: *H′* = −Σ*p_i_* ln *p_i_*, where, *p_i_* is the proportion of seeds from species *i* relative to the total number of seeds in a given fecal sample.

The data I used in this study is given in Table S1.

### Statistical analysis

To evaluate the variation in the *AR*, I fitted a generalized linear model (GLM) in which the response variable was binary (feces containing seeds or not, independently of plant species; binomial error family) and the fixed effect was year and month. I performed chi-square tests for independence to examine inter-annual changes in the *AR* in October for each fleshy-fruited species separately. To evaluate the differences in the *NS*, I ran another GLM with count data (negative binomial error family). Only feces containing seeds were included in this model. The response variable was the total number of seeds found in each fecal sample, and the explanatory variables were year, month, and “dominant plant species” (*DPS* hereafter), defined as the most abundant seed species in a fecal sample [2]. In this analysis, I also considered the effect of interaction between year, month, and the *DPS*. In order to test the differences in the *IR*, I fitted another GLM with the negative binomial error family. The response variable was the number of intact seeds in each fecal sample (overall number of seeds were treated as an offset term), and the explanatory factors were the same as the second GLM model. Finally, to analyze the seed diversity in feces, I again ran GLM (Gamma error family) with seed diversity as a response variable and the fixed effects were the same as those in the second and third GLM models. I performed these GLMs using the “MASS” and “multcomp” packages in the statistical software R.15.2. [41]. The significance levels of these analyses were set at 5%.

## Supporting Information

Table S1 **A.** Number of all seeds detected from fecal analyses collected during five study years. Seed diversity has been calculated at the Shannon Winer index (*H'*). **B.** Number of intact seeds detected from fecal samples collected during five study years. Intact ratio (*IR*) is also shown.
(DOC)
