# Supplementary material for: Inter-Annual Variation in Characteristics of Endozoochory by Wild Japanese Macaques
Source: PLoS One. 2014 Oct 1;9(10):e108155. doi: 10.1371/journal.pone.0108155 (PMC4182713; doi:10.1371/journal.pone.0108155)
Supplement: Table S1 — A. Number of all seeds detected from fecal analyses collected during five study years. Seed diversity has been calculated at the Shannon Winer index (H'). B. Number of intact seeds detected from fecal samples collected during five study years. Intact ratio (IR) is also shown. (DOC) [file pone.0108155.s001.doc]

**Table S1**

a.

| Sample | Year | Month | *DPS* | Species | | | | | | | | | | | | | | | | Total | *H'* |
| --- | --- | --- | --- | --- | --- | --- | --- | --- | --- | --- | --- | --- | --- | --- | --- | --- | --- | --- | --- | --- | --- |
|  |  |  |  | 1 | 2 | 3 | 4 | 5 | 6 | 7 | 9 | 13 | 14 | 16 | 17 | 18 | 19 | 21 | X |  |  |
| KI0409001 | 2004 | Sep | 14 |  |  |  |  |  |  |  |  |  | 1 |  |  |  |  |  |  | 1 | 0.001 |
| KI0409002 | 2004 | Sep | 2 |  | 7 |  |  |  |  |  |  |  |  |  |  |  |  |  |  | 7 | 0.001 |
| KI0409003 | 2004 | Sep | 2 |  | 2 |  |  |  |  |  |  |  |  |  |  |  |  |  |  | 2 | 0.001 |
| KI0509001 | 2005 | Sep | 16 |  |  |  |  |  |  |  |  |  |  | 62 | 29 |  | 22 |  |  | 113 | 0.997 |
| KI0509002 | 2005 | Sep |  |  |  |  |  |  |  |  |  |  |  |  |  |  |  |  |  | 0 | - |
| KI0509003 | 2005 | Sep | 18 |  |  |  |  |  |  |  |  |  |  | 30 | 25 | 61 |  |  |  | 116 | 1.018 |
| KI0509004 | 2005 | Sep | 16 |  |  |  |  |  |  |  |  |  |  | 1 |  |  |  |  |  | 1 | 0.001 |
| KI0509005 | 2005 | Sep | 16 |  |  |  |  |  |  |  |  |  |  | 5 |  |  |  |  |  | 5 | 0.001 |
| KI0509006 | 2005 | Sep | 16 |  |  |  |  |  | 3 |  |  |  |  | 49 | 14 |  |  |  |  | 66 | 0.691 |
| KI0509007 | 2005 | Sep | 18 |  |  |  |  |  |  |  |  |  |  | 35 | 26 | 52 |  |  |  | 113 | 1.058 |
| KI0509008 | 2005 | Sep |  |  |  |  |  |  |  |  |  |  |  |  |  |  |  |  |  | 0 | - |
| KI0509009 | 2005 | Sep | 16 |  |  |  |  |  |  |  |  |  |  | 1 |  |  |  |  |  | 1 | 0.001 |
| KI0509010 | 2005 | Sep | 16 |  |  |  |  |  |  |  |  |  |  | 27 |  |  |  |  |  | 27 | 0.001 |
| KI0509011 | 2005 | Sep | 16 |  |  |  |  |  |  |  |  |  |  | 4 |  |  |  |  |  | 4 | 0.001 |
| KI0709001 | 2007 | Sep | 17 |  |  |  |  |  |  |  |  |  |  |  | 4 |  |  |  |  | 4 | 0.001 |
| KI0709002 | 2007 | Sep | 17 |  |  |  |  |  |  |  |  |  |  | 1 | 62 |  | 5 |  |  | 68 | 0.338 |
| KI0709003 | 2007 | Sep | 18 |  |  |  |  |  |  |  |  |  |  |  |  | 56 |  |  |  | 56 | 0.001 |
| KI0709004 | 2007 | Sep | 17 |  |  |  |  |  |  | 1 |  |  |  |  | 18 |  | 7 |  |  | 26 | 0.864 |
| KI0709005 | 2007 | Sep | 17 |  |  |  |  |  |  | 1 |  |  |  |  | 7 |  | 2 |  |  | 10 | 0.802 |
| KI0709006 | 2007 | Sep | 7 |  |  |  |  |  |  | 29 |  |  |  |  |  |  | 26 |  |  | 55 | 0.692 |
| KI0709007 | 2007 | Sep |  |  |  |  |  |  |  |  |  |  |  |  |  |  |  |  |  | 0 | - |
| KI0709008 | 2007 | Sep | 19 |  |  |  |  |  |  |  |  |  |  |  |  |  | 44 |  |  | 44 | 0.001 |
| KI0709009 | 2007 | Sep | 7 |  |  |  |  |  |  | 4 |  |  |  |  | 3 |  |  |  |  | 7 | 0.683 |
| KI0709010 | 2007 | Sep | 17 |  |  |  |  |  |  | 2 |  |  |  | 1 | 77 |  |  |  |  | 80 | 0.184 |
| KI0709011 | 2007 | Sep | 7 |  |  |  |  |  |  | 13 |  |  |  |  | 5 |  | 8 |  |  | 26 | 1.026 |
| KI0709012 | 2007 | Sep | 19 |  |  |  |  |  |  | 3 |  |  |  |  |  |  | 19 |  |  | 22 | 0.398 |
| KI0709013 | 2007 | Sep | 7 |  |  |  |  |  |  | 3 |  |  |  |  |  |  | 2 |  |  | 5 | 0.673 |
| KI0709014 | 2007 | Sep | 5 |  |  |  |  | 33 |  | 5 |  |  |  |  | 18 |  | 1 |  |  | 57 | 0.965 |
| KI0709015 | 2007 | Sep | 19 |  |  |  |  |  |  |  |  |  |  |  |  |  | 51 |  |  | 51 | 0.001 |
| KI0709016 | 2007 | Sep | 17 |  |  |  |  |  |  |  |  |  |  |  | 26 |  | 8 |  |  | 34 | 0.546 |
| KI0709017 | 2007 | Sep | 17 |  |  |  |  |  |  | 1 |  |  |  |  | 65 |  | 18 |  |  | 84 | 0.581 |
| KI0709018 | 2007 | Sep | 17 |  |  |  |  |  |  |  |  |  |  |  | 140 |  | 5 |  |  | 145 | 0.150 |
| KI0709019 | 2007 | Sep |  |  |  |  |  |  |  |  |  |  |  |  |  |  |  |  |  | 0 | - |
| KI0709020 | 2007 | Sep | 17 |  |  |  |  |  |  | 2 |  |  |  |  | 10 |  |  |  |  | 12 | 0.451 |
| KI0709021 | 2007 | Sep | 1 | 157 | 1 |  |  |  |  | 1 |  |  |  | 1 |  |  | 98 |  |  | 258 | 0.735 |
| KI0709022 | 2007 | Sep |  |  |  |  |  |  |  |  |  |  |  |  |  |  |  |  |  | 0 | - |
| KI0709023 | 2007 | Sep | 19 |  | 2 |  |  |  |  |  |  |  |  |  |  |  | 26 |  |  | 28 | 0.257 |
| KI0709024 | 2007 | Sep | 19 |  |  |  |  |  |  |  |  |  |  |  | 2 |  | 6 |  |  | 8 | 0.562 |
| KI0709025 | 2007 | Sep | 17 |  |  |  |  |  |  | 1 |  |  |  |  | 6 |  | 3 |  |  | 10 | 0.898 |
| KI0709026 | 2007 | Sep | 19 |  |  |  |  |  |  |  |  |  |  |  | 3 |  | 11 |  |  | 14 | 0.520 |
| KI0709027 | 2007 | Sep | 19 |  |  |  |  |  |  | 1 |  |  |  |  |  |  | 134 |  |  | 135 | 0.044 |
| KI0709028 | 2007 | Sep | 19 |  |  |  |  |  |  | 1 |  |  |  |  | 6 |  | 29 |  |  | 36 | 0.572 |
| KI0709029 | 2007 | Sep | 17 |  |  |  |  |  |  | 2 |  |  |  |  | 29 |  | 16 |  |  | 47 | 0.799 |
| KI0809001 | 2008 | Sep | 17 |  |  |  |  |  |  |  |  |  |  |  | 89 |  |  |  |  | 89 | 0.001 |
| KI0809002 | 2008 | Sep | 17 |  |  |  |  |  |  |  |  |  |  | 4 | 61 |  |  |  |  | 65 | 0.231 |
| KI0809003 | 2008 | Sep | 17 |  | 4 |  |  |  |  |  |  |  |  | 2 | 42 |  |  |  |  | 48 | 0.456 |
| KI0809004 | 2008 | Sep | 16 |  | 2 |  |  |  |  |  |  |  |  | 41 | 7 | 2 |  |  |  | 52 | 0.708 |
| KI0809005 | 2008 | Sep | 17 |  |  |  |  |  |  |  |  |  |  | 13 | 41 |  |  |  |  | 54 | 0.552 |
| KI0809006 | 2008 | Sep | 17 |  |  |  |  |  |  |  |  |  |  | 1 | 8 |  |  |  |  | 9 | 0.349 |
| KI0809007 | 2008 | Sep | 16 |  | 3 |  |  |  |  |  |  |  |  | 32 | 3 |  |  |  |  | 38 | 0.546 |
| KI0809008 | 2008 | Sep | 17 |  |  |  |  |  |  |  |  |  |  |  | 44 |  | 1 |  |  | 45 | 0.107 |
| KI0809009 | 2008 | Sep | 16 |  |  |  |  |  |  | 1 |  |  |  | 27 | 2 | 1 |  |  |  | 31 | 0.519 |
| KI0809010 | 2008 | Sep | 16 |  | 1 |  |  |  |  |  |  |  |  | 36 | 19 |  |  |  |  | 56 | 0.723 |
| KI0809011 | 2008 | Sep | 16 |  |  |  |  |  |  |  |  |  |  | 82 |  |  |  |  |  | 82 | 0.001 |
| KI0809012 | 2008 | Sep | 2 |  | 5 |  |  |  |  |  |  |  |  | 162 | 48 |  |  |  |  | 215 | 0.635 |
| KI0809013 | 2008 | Sep | 17 |  |  |  |  |  |  |  |  |  |  | 6 | 14 |  |  |  |  | 20 | 0.611 |
| KI0809014 | 2008 | Sep | 16 |  | 1 |  |  |  |  |  |  |  |  | 57 | 15 |  |  |  |  | 73 | 0.577 |
| KI0809015 | 2008 | Sep | 17 |  |  |  |  |  |  |  |  |  |  | 10 | 62 |  |  |  |  | 72 | 0.403 |
| KI0809016 | 2008 | Sep | 17 |  |  |  |  |  |  | 2 |  |  |  | 11 | 44 |  |  |  |  | 57 | 0.635 |
| KI0809017 | 2008 | Sep | 17 |  |  |  |  |  |  |  |  |  |  | 30 | 90 |  | 2 |  |  | 122 | 0.637 |
| KI0809018 | 2008 | Sep | 17 |  | 2 |  |  |  |  |  |  |  |  |  | 103 |  | 1 |  |  | 106 | 0.147 |
| KI0809019 | 2008 | Sep |  |  |  |  |  |  |  |  |  |  |  |  |  |  |  |  |  | 0 | - |
| KI0809020 | 2008 | Sep |  |  |  |  |  |  |  |  |  |  |  |  |  |  |  |  |  | 0 | - |
| KI0809021 | 2008 | Sep |  |  |  |  |  |  |  |  |  |  |  |  |  |  |  |  |  | 0 | - |
| KI0809022 | 2008 | Sep |  |  |  |  |  |  |  |  |  |  |  |  |  |  |  |  |  | 0 | - |
| KI0809023 | 2008 | Sep |  |  |  |  |  |  |  |  |  |  |  |  |  |  |  |  |  | 0 | - |
| KI0809024 | 2008 | Sep |  |  |  |  |  |  |  |  |  |  |  |  |  |  |  |  |  | 0 | - |
| KI0809025 | 2008 | Sep |  |  |  |  |  |  |  |  |  |  |  |  |  |  |  |  |  | 0 | - |
| KI0809026 | 2008 | Sep |  |  |  |  |  |  |  |  |  |  |  |  |  |  |  |  |  | 0 | - |
| KI0809027 | 2008 | Sep |  |  |  |  |  |  |  |  |  |  |  |  |  |  |  |  |  | 0 | - |
| KI0809028 | 2008 | Sep |  |  |  |  |  |  |  |  |  |  |  |  |  |  |  |  |  | 0 | - |
| KI0809029 | 2008 | Sep |  |  |  |  |  |  |  |  |  |  |  |  |  |  |  |  |  | 0 | - |
| KI0809030 | 2008 | Sep |  |  |  |  |  |  |  |  |  |  |  |  |  |  |  |  |  | 0 | - |
| KI0809031 | 2008 | Sep |  |  |  |  |  |  |  |  |  |  |  |  |  |  |  |  |  | 0 | - |
| KI0809032 | 2008 | Sep |  |  |  |  |  |  |  |  |  |  |  |  |  |  |  |  |  | 0 | - |
| KI0809033 | 2008 | Sep |  |  |  |  |  |  |  |  |  |  |  |  |  |  |  |  |  | 0 | - |
| KI0809034 | 2008 | Sep |  |  |  |  |  |  |  |  |  |  |  |  |  |  |  |  |  | 0 | - |
| KI0809035 | 2008 | Sep |  |  |  |  |  |  |  |  |  |  |  |  |  |  |  |  |  | 0 | - |
| KI0809036 | 2008 | Sep |  |  |  |  |  |  |  |  |  |  |  |  |  |  |  |  |  | 0 | - |
| KI0010001 | 2000 | Oct | 1 | 4 |  |  |  |  |  |  |  |  |  |  |  |  |  |  |  | 4 | 0.001 |
| KI0010002 | 2000 | Oct | 2 |  | 7 |  |  |  |  |  |  |  |  |  |  |  |  |  |  | 7 | 0.001 |
| KI0010003 | 2000 | Oct | 2 |  | 30 |  |  |  |  |  |  |  |  |  |  |  |  |  |  | 30 | 0.001 |
| KI0010004 | 2000 | Oct |  |  |  |  |  |  |  |  |  |  |  |  |  |  |  |  |  | 0 | - |
| KI0010005 | 2000 | Oct | 25 |  |  |  |  | 1 |  |  |  |  |  |  |  |  |  | 1 |  | 2 | 0.693 |
| KI0010006 | 2000 | Oct | 2 |  | 1 |  |  |  |  |  |  |  |  |  |  |  |  |  |  | 1 | 0.001 |
| KI0010007 | 2000 | Oct | 2 |  | 1 |  |  |  |  |  |  |  |  |  |  |  |  |  |  | 1 | 0.001 |
| KI0010008 | 2000 | Oct | 2 |  | 2 |  |  |  |  |  |  |  |  |  |  |  |  |  |  | 2 | 0.001 |
| KI0010009 | 2000 | Oct |  |  |  |  |  |  |  |  |  |  |  |  |  |  |  |  |  | 0 | - |
| KI0010010 | 2000 | Oct |  |  |  |  |  |  |  |  |  |  |  |  |  |  |  |  |  | 0 | - |
| KI0010011 | 2000 | Oct | 2 | 2 | 3 |  | 1 |  | 1 |  |  |  |  |  |  |  |  |  |  | 7 | 1.277 |
| KI0010012 | 2000 | Oct | 2 |  | 23 |  |  |  |  |  |  |  |  |  |  |  |  |  |  | 23 | 0.001 |
| KI0010013 | 2000 | Oct | 6 |  |  |  |  |  | 56 |  |  |  |  |  |  |  |  |  |  | 56 | 0.001 |
| KI0010014 | 2000 | Oct | 2 |  | 37 | 1 |  |  | 1 |  |  |  |  |  |  |  |  |  |  | 39 | 0.238 |
| KI0010015 | 2000 | Oct | 1 | 49 | 19 |  |  |  | 12 |  |  |  |  |  |  |  |  |  |  | 80 | 0.926 |
| KI0010016 | 2000 | Oct | 2 | 1 | 17 |  |  |  |  |  |  |  |  |  |  |  |  |  |  | 18 | 0.409 |
| KI0010017 | 2000 | Oct | 6 |  |  |  |  |  | 4 |  |  |  |  |  |  |  |  |  |  | 4 | 0.001 |
| KI0010018 | 2000 | Oct | 3 | 1 | 6 | 10 |  |  |  |  |  |  |  |  |  |  |  |  |  | 17 | 0.846 |
| KI0010019 | 2000 | Oct | 2 | 8 | 60 |  |  | 9 | 12 |  |  |  |  |  |  |  |  |  |  | 89 | 0.984 |
| KI0010020 | 2000 | Oct | 3 |  |  | 9 |  |  |  |  |  |  |  |  |  |  |  |  |  | 9 | 0.001 |
| KI0010021 | 2000 | Oct | 2 |  | 15 |  |  |  | 6 |  |  |  |  |  |  |  |  |  |  | 21 | 0.598 |
| KI0010022 | 2000 | Oct | 1 | 106 | 50 | 5 |  |  | 1 | 1 |  |  |  |  |  |  |  |  |  | 163 | 0.812 |
| KI0010023 | 2000 | Oct |  |  |  |  |  |  |  |  |  |  |  |  |  |  |  |  |  | 0 | - |
| KI0010024 | 2000 | Oct | 5 |  |  |  |  | 5 |  |  |  |  |  |  |  |  |  |  |  | 5 | 0.451 |
| KI0010025 | 2000 | Oct | 2 |  | 17 |  |  | 14 |  |  |  |  | 1 |  |  |  |  |  |  | 32 | 0.806 |
| KI0010026 | 2000 | Oct | 2 |  | 12 |  |  | 6 | 5 |  |  |  |  |  |  |  |  |  |  | 23 | 1.022 |
| KI0010027 | 2000 | Oct | 2 |  | 30 | 1 |  | 5 | 8 |  |  |  |  |  |  |  |  |  |  | 44 | 0.904 |
| KI0010028 | 2000 | Oct | 2 | 1 | 37 |  |  |  | 8 |  |  |  | 1 |  |  |  |  |  |  | 47 | 0.654 |
| KI0010029 | 2000 | Oct | 6 |  | 9 |  |  | 3 | 11 |  |  |  | 1 |  |  |  |  |  |  | 24 | 1.118 |
| KI0010030 | 2000 | Oct | 3 |  |  | 8 |  |  |  |  |  |  |  |  |  |  |  |  |  | 8 | 0.001 |
| KI0010031 | 2000 | Oct | 1 | 372 | 52 | 5 |  |  | 5 | 1 |  |  |  |  |  |  |  | 1 |  | 436 | 0.519 |
| KI0010032 | 2000 | Oct | 2 | 19 | 21 |  |  |  | 3 |  |  |  |  |  |  |  |  |  |  | 43 | 0.897 |
| KI0010033 | 2000 | Oct | 25 |  | 1 |  |  |  | 1 |  |  |  |  |  |  |  |  |  |  | 2 | 0.693 |
| KI0010034 | 2000 | Oct | 6 |  |  |  |  |  | 8 |  |  |  |  |  |  |  |  |  |  | 8 | 0.001 |
| KI0010035 | 2000 | Oct | 25 |  | 5 | 1 |  |  | 5 |  |  |  |  |  |  |  |  |  |  | 11 | 0.935 |
| KI0010036 | 2000 | Oct | 6 |  |  |  |  |  | 1 |  |  |  |  |  |  |  |  |  |  | 1 | 0.001 |
| KI0010037 | 2000 | Oct |  |  |  |  |  |  |  |  |  |  |  |  |  |  |  |  |  | 0 | - |
| KI0010038 | 2000 | Oct | 6 | 1 | 4 |  |  |  | 10 |  |  |  |  |  |  |  |  |  |  | 15 | 0.803 |
| KI0010039 | 2000 | Oct | 2 |  | 25 |  |  | 3 | 7 |  | 1 |  |  |  |  |  |  |  |  | 36 | 0.878 |
| KI0010040 | 2000 | Oct | 1 | 29 |  |  |  | 4 | 1 |  |  |  |  |  | 1 |  |  |  |  | 35 | 0.607 |
| KI0010041 | 2000 | Oct | 4 |  | 5 | 1 | 47 |  |  |  |  |  |  |  |  |  |  |  |  | 53 | 0.404 |
| KI0010042 | 2000 | Oct | 2 |  | 14 | 7 | 1 |  | 3 |  | 2 |  |  |  |  |  |  |  |  | 27 | 1.250 |
| KI0010043 | 2000 | Oct | 2 |  | 27 | 1 |  |  | 10 |  |  |  |  |  |  |  |  |  |  | 38 | 0.690 |
| KI0010044 | 2000 | Oct | 6 |  |  |  |  |  | 10 |  |  |  |  |  |  |  |  |  |  | 10 | 0.001 |
| KI0010045 | 2000 | Oct | 6 | 4 | 6 |  |  | 2 | 8 |  |  |  | 2 |  |  |  |  |  |  | 22 | 1.468 |
| KI0010046 | 2000 | Oct | 25 |  | 2 |  |  |  | 2 |  | 1 |  |  |  |  |  |  | 1 |  | 6 | 1.550 |
| KI0010047 | 2000 | Oct | 1 | 13 | 10 |  |  |  |  |  |  |  |  |  |  |  |  |  |  | 23 | 0.829 |
| KI0010048 | 2000 | Oct |  |  |  |  |  |  |  |  |  |  |  |  |  |  |  | 1 |  | 1 | 0.001 |
| KI0010049 | 2000 | Oct | 2 | 2 | 40 |  |  |  | 1 |  |  |  | 5 |  |  |  |  | 4 |  | 52 | 0.826 |
| KI0010050 | 2000 | Oct | 25 |  |  |  |  | 1 |  |  |  |  |  |  |  |  |  |  |  | 1 | 0.693 |
| KI0010051 | 2000 | Oct | 6 | 1 | 10 |  | 1 |  | 14 |  |  |  |  |  |  |  |  | 4 |  | 30 | 1.217 |
| KI0010052 | 2000 | Oct | 5 |  | 6 |  |  | 15 | 1 |  |  |  | 2 |  |  |  |  | 22 |  | 46 | 1.203 |
| KI0010053 | 2000 | Oct | 5 | 1 | 2 |  | 11 | 44 | 8 |  |  |  |  |  |  |  |  |  |  | 66 | 0.994 |
| KI0010054 | 2000 | Oct |  |  |  |  |  |  |  |  |  |  |  |  |  |  |  |  |  | 0 | - |
| KI0010055 | 2000 | Oct | 5 |  |  |  |  | 10 | 2 |  |  |  |  |  |  |  |  |  |  | 12 | 0.796 |
| KI0010056 | 2000 | Oct | 2 |  | 24 |  | 9 | 6 | 1 |  |  |  |  |  |  |  |  |  |  | 40 | 1.019 |
| KI0010057 | 2000 | Oct | 6 |  | 2 |  |  |  | 10 |  |  |  |  |  |  |  |  |  |  | 12 | 0.451 |
| KI0010058 | 2000 | Oct |  |  |  |  |  |  |  |  |  |  |  |  |  |  |  |  |  | 0 | - |
| KI0010059 | 2000 | Oct | 21 |  |  |  |  | 5 |  |  |  |  |  |  |  | 1 |  | 13 |  | 19 | 0.766 |
| KI0010060 | 2000 | Oct | 6 |  | 3 |  |  |  | 13 |  |  |  |  |  |  |  |  |  |  | 16 | 0.483 |
| KI0010061 | 2000 | Oct |  |  |  |  |  |  |  |  |  |  |  |  |  |  |  |  |  | 0 | - |
| KI0010062 | 2000 | Oct | 6 |  | 6 |  |  |  | 124 |  |  |  |  |  |  |  |  |  |  | 130 | 0.187 |
| KI0010063 | 2000 | Oct | 5 |  | 1 |  |  | 10 | 2 |  |  |  |  |  |  |  |  |  |  | 13 | 0.895 |
| KI0010064 | 2000 | Oct | 2 |  | 19 |  |  |  | 4 |  |  |  |  |  |  |  |  | 7 |  | 30 | 0.898 |
| KI0010065 | 2000 | Oct | 4 |  |  |  | 6 | 5 |  |  |  |  |  |  |  |  |  |  |  | 11 | 0.689 |
| KI0010066 | 2000 | Oct | 6 |  | 6 |  |  |  | 70 |  | 2 |  |  |  |  |  |  |  |  | 78 | 0.388 |
| KI0410001 | 2004 | Oct | 4 |  |  |  | 42 |  |  |  |  |  |  |  |  |  |  |  |  | 42 | 0.001 |
| KI0410002 | 2004 | Oct | 4 |  |  |  | 13 |  |  |  |  |  |  |  |  |  | 1 |  |  | 14 | 0.257 |
| KI0410003 | 2004 | Oct | 4 |  |  |  | 164 |  |  |  |  |  |  |  |  |  |  |  |  | 164 | 0.001 |
| KI0410004 | 2004 | Oct | 4 |  |  |  | 2 |  |  |  |  |  |  |  |  |  |  |  |  | 2 | 0.001 |
| KI0410005 | 2004 | Oct | 4 |  |  |  | 3 |  |  |  |  |  |  |  |  |  |  |  |  | 3 | 0.001 |
| KI0410006 | 2004 | Oct | 4 |  |  |  | 76 |  |  |  |  |  |  |  |  |  |  |  |  | 76 | 0.001 |
| KI0410007 | 2004 | Oct | 17 |  |  |  | 6 |  |  |  |  |  |  |  | 10 |  |  |  |  | 16 | 0.846 |
| KI0410008 | 2004 | Oct | 4 | 5 |  | 84 | 110 |  |  |  |  |  |  |  |  |  |  |  |  | 199 | 0.784 |
| KI0410009 | 2004 | Oct | 4 |  |  |  | 39 |  |  |  |  |  |  |  |  |  |  |  |  | 39 | 0.001 |
| KI0410010 | 2004 | Oct | 4 |  |  | 2 | 27 |  |  |  |  |  |  |  |  |  |  |  |  | 29 | 0.251 |
| KI0410011 | 2004 | Oct | 3 |  |  | 6 | 1 |  |  |  |  |  |  |  |  |  |  |  |  | 7 | 0.410 |
| KI0410012 | 2004 | Oct | 4 |  |  |  | 10 |  |  |  |  |  |  |  |  |  |  |  |  | 10 | 0.001 |
| KI0410013 | 2004 | Oct | 25 |  |  |  | 1 |  |  |  |  |  | 1 |  |  |  |  |  |  | 2 | 1.099 |
| KI0410014 | 2004 | Oct | 4 | 1 |  |  | 91 |  |  |  | 54 |  | 4 |  |  |  |  |  |  | 150 | 0.836 |
| KI0410015 | 2004 | Oct | 4 |  |  | 72 | 640 |  | 1 |  |  |  |  |  |  |  |  |  |  | 713 | 0.348 |
| KI0410016 | 2004 | Oct |  |  |  |  |  |  |  |  |  |  |  |  |  |  |  |  |  | 0 | - |
| KI0410017 | 2004 | Oct |  |  |  |  |  |  |  |  |  |  |  |  |  |  |  |  |  | 0 | - |
| KI0410018 | 2004 | Oct | 4 |  |  | 1 | 26 |  |  |  | 1 |  |  |  |  |  |  |  |  | 28 | 0.307 |
| KI0410019 | 2004 | Oct | 4 |  |  | 17 | 23 |  |  |  |  |  |  |  |  |  |  |  |  | 40 | 0.682 |
| KI0410020 | 2004 | Oct | 4 |  |  |  | 92 |  |  |  | 1 |  | 4 |  |  |  |  |  |  | 97 | 0.229 |
| KI0410021 | 2004 | Oct | 3 |  |  | 268 | 164 |  |  |  | 4 |  |  |  |  |  |  |  |  | 436 | 0.710 |
| KI0410022 | 2004 | Oct |  |  |  |  |  |  |  |  |  |  |  |  |  |  |  |  |  | 0 | - |
| KI0410023 | 2004 | Oct | 4 |  |  |  | 22 |  |  |  | 2 |  | 3 |  |  |  |  |  |  | 27 | 0.604 |
| KI0410024 | 2004 | Oct | 4 |  |  |  | 156 |  |  |  |  |  |  |  |  |  |  |  |  | 156 | 0.001 |
| KI0510001 | 2005 | Oct |  |  |  |  |  |  |  |  |  |  |  |  |  |  | 3 |  |  | 3 | 0.001 |
| KI0510002 | 2005 | Oct | 4 |  |  |  | 40 |  |  |  |  |  |  |  |  |  |  |  |  | 40 | 0.001 |
| KI0510003 | 2005 | Oct | 17 |  |  | 1 |  |  |  |  |  |  |  | 16 | 39 | 74 |  |  |  | 130 | 0.977 |
| KI0510004 | 2005 | Oct | 16 |  |  |  |  |  |  |  |  |  |  | 17 | 13 |  |  |  |  | 30 | 0.684 |
| KI0510005 | 2005 | Oct | 4 |  |  |  | 40 |  |  |  |  |  |  |  | 1 |  | 17 |  |  | 58 | 0.686 |
| KI0510006 | 2005 | Oct | 4 |  |  |  | 45 |  |  |  |  |  |  |  |  |  |  |  |  | 45 | 0.001 |
| KI0510007 | 2005 | Oct | 4 |  |  |  | 51 |  |  |  |  |  |  |  |  |  | 1 |  |  | 52 | 0.095 |
| KI0510008 | 2005 | Oct | 19 |  |  |  | 1 |  |  |  |  |  |  |  | 10 |  | 24 |  |  | 35 | 0.718 |
| KI0510009 | 2005 | Oct | 17 |  |  |  |  |  |  |  |  |  |  |  | 8 |  | 3 |  |  | 11 | 0.586 |
| KI0510010 | 2005 | Oct | 17 |  |  |  |  |  |  |  |  |  |  | 1 | 11 |  | 4 |  |  | 16 | 0.777 |
| KI0510011 | 2005 | Oct | 25 |  |  |  | 4 |  |  |  |  |  |  | 7 | 3 |  | 7 |  |  | 21 | 1.326 |
| KI0510012 | 2005 | Oct | 19 |  |  |  |  |  |  |  |  |  |  |  | 1 |  | 48 |  |  | 49 | 0.100 |
| KI0510013 | 2005 | Oct | 19 |  |  |  | 14 |  |  |  |  |  |  |  | 6 |  | 36 |  |  | 56 | 0.870 |
| KI0510014 | 2005 | Oct | 19 |  |  |  |  |  |  |  |  |  |  |  |  |  | 11 |  |  | 11 | 0.001 |
| KI0510015 | 2005 | Oct | 4 |  | 1 |  | 6 |  | 1 |  |  |  |  |  | 2 |  | 3 |  |  | 13 | 1.378 |
| KI0510016 | 2005 | Oct | 6 |  |  |  |  |  | 20 |  |  |  |  |  |  |  |  |  |  | 20 | 0.001 |
| KI0510017 | 2005 | Oct | 17 |  |  |  |  |  |  |  |  |  |  | 2 | 8 |  |  |  |  | 10 | 0.500 |
| KI0510018 | 2005 | Oct | 17 |  |  |  |  |  | 1 |  |  |  |  |  | 2 |  | 1 |  |  | 4 | 1.040 |
| KI0510019 | 2005 | Oct | 17 |  |  |  | 4 |  |  |  |  |  |  |  | 33 |  | 14 |  |  | 51 | 0.836 |
| KI0510020 | 2005 | Oct | 19 |  |  |  | 3 |  |  |  |  |  |  |  | 23 |  | 38 |  |  | 64 | 0.821 |
| KI0510021 | 2005 | Oct | 19 |  |  |  |  |  |  |  |  |  |  |  |  |  | 4 |  |  | 4 | 0.001 |
| KI0510022 | 2005 | Oct | 4 |  |  |  | 33 |  |  |  |  |  |  |  | 1 |  | 3 |  |  | 37 | 0.514 |
| KI0510023 | 2005 | Oct | 4 |  |  |  | 70 |  | 3 |  |  |  |  |  | 1 |  |  |  |  | 74 | 0.241 |
| KI0510024 | 2005 | Oct | 4 |  |  |  | 155 |  | 16 |  |  |  |  |  | 14 |  |  |  |  | 185 | 0.555 |
| KI0510025 | 2005 | Oct | 4 |  |  |  | 1 |  |  |  |  |  |  |  |  |  |  |  |  | 1 | 0.001 |
| KI0510026 | 2005 | Oct | 17 |  |  |  |  |  |  |  |  |  |  | 3 | 43 |  |  |  |  | 46 | 0.241 |
| KI0510027 | 2005 | Oct | 4 |  |  |  | 65 |  |  | 1 |  |  |  |  |  |  |  |  |  | 66 | 0.079 |
| KI0510028 | 2005 | Oct | 19 |  |  |  | 1 |  |  |  |  |  |  |  | 1 |  | 12 |  |  | 14 | 0.509 |
| KI0510029 | 2005 | Oct | 17 |  | 3 |  | 2 |  |  |  |  |  |  |  | 92 |  | 11 |  |  | 108 | 0.543 |
| KI0510030 | 2005 | Oct | 19 |  |  |  | 6 |  |  |  |  |  |  |  |  |  | 40 |  |  | 46 | 0.387 |
| KI0510031 | 2005 | Oct | 19 |  |  |  |  |  |  |  |  |  |  |  |  |  | 2 |  |  | 2 | 0.001 |
| KI0710001 | 2007 | Oct | 4 |  |  |  | 25 |  | 23 |  |  |  |  |  |  |  |  |  |  | 48 | 0.692 |
| KI0710002 | 2007 | Oct | 1 | 290 |  | 4 | 13 |  | 30 |  |  |  |  |  |  |  |  |  |  | 337 | 0.523 |
| KI0710003 | 2007 | Oct | 4 |  |  |  | 33 |  | 24 | 1 |  |  |  |  |  |  |  |  |  | 58 | 0.756 |
| KI0710004 | 2007 | Oct | 6 |  | 7 |  | 1 |  | 15 |  |  |  |  |  |  |  |  |  |  | 23 | 0.777 |
| KI0710005 | 2007 | Oct | 4 |  |  |  | 25 |  | 13 | 2 |  |  |  |  |  |  |  |  |  | 40 | 0.809 |
| KI0710006 | 2007 | Oct | 3 |  | 1 | 61 | 31 |  | 18 |  |  |  |  |  |  |  |  |  |  | 111 | 1.023 |
| KI0710007 | 2007 | Oct | 6 |  |  |  |  |  | 45 | 1 | 35 |  |  |  |  |  |  |  |  | 81 | 0.743 |
| KI0710008 | 2007 | Oct | 4 |  | 1 |  | 44 |  | 21 |  | 14 |  |  |  |  |  | 2 |  |  | 82 | 1.129 |
| KI0710009 | 2007 | Oct | 4 |  |  |  | 21 |  | 19 |  |  |  |  |  |  |  |  |  |  | 40 | 0.692 |
| KI0710010 | 2007 | Oct | 4 |  |  |  | 46 |  | 1 |  |  |  |  |  |  |  |  |  |  | 47 | 0.103 |
| KI0710011 | 2007 | Oct | 6 | 81 | 13 |  | 31 |  | 88 | 8 | 12 |  |  |  |  |  |  |  |  | 233 | 1.433 |
| KI0710012 | 2007 | Oct | 4 |  |  |  | 134 |  | 12 |  |  |  |  |  |  |  |  |  |  | 146 | 0.284 |
| KI0710013 | 2007 | Oct | 4 |  | 1 | 1 | 117 |  | 2 |  | 2 |  |  |  |  |  |  |  |  | 123 | 0.260 |
| KI0710014 | 2007 | Oct | 4 |  |  |  | 25 |  | 15 | 3 |  |  |  |  |  |  |  |  |  | 43 | 0.868 |
| KI0710015 | 2007 | Oct | 6 |  |  | 1 | 12 |  | 126 | 4 |  |  |  |  |  |  |  |  |  | 143 | 0.492 |
| KI0710016 | 2007 | Oct | 4 |  | 2 |  | 125 |  | 40 | 3 | 1 |  | 1 |  | 4 |  |  |  |  | 176 | 0.845 |
| KI0710017 | 2007 | Oct | 6 |  | 1 |  |  |  | 44 | 1 | 12 |  |  |  |  |  |  |  |  | 58 | 0.676 |
| KI0710018 | 2007 | Oct | 4 |  | 4 |  | 56 |  | 27 | 2 |  |  |  |  |  |  |  |  |  | 89 | 0.878 |
| KI0710019 | 2007 | Oct | 4 |  |  |  | 18 |  | 4 | 1 |  |  |  |  |  |  |  |  |  | 23 | 0.632 |
| KI0710020 | 2007 | Oct | 6 |  |  |  | 3 |  | 9 |  | 5 |  |  |  |  |  |  |  |  | 17 | 1.003 |
| KI0710021 | 2007 | Oct | 6 |  |  |  | 6 |  | 67 |  | 22 |  |  |  |  |  |  |  |  | 95 | 0.759 |
| KI0710022 | 2007 | Oct | 6 |  | 1 | 4 | 6 |  | 9 |  | 2 |  |  |  |  |  |  |  |  | 22 | 1.388 |
| KI0710023 | 2007 | Oct | 6 |  |  | 5 | 54 | 28 | 90 |  | 1 |  |  |  |  |  |  |  |  | 178 | 1.155 |
| KI0710024 | 2007 | Oct | 6 |  | 1 |  |  |  | 88 |  | 1 |  |  |  |  |  |  |  |  | 90 | 0.122 |
| KI0710025 | 2007 | Oct | 4 |  | 2 | 1 | 101 |  | 11 | 6 |  |  |  |  |  |  |  |  |  | 121 | 0.625 |
| KI0810001 | 2008 | Oct | 4 |  | 1 |  | 5 |  |  |  |  |  |  |  |  |  | 1 |  |  | 7 | 0.796 |
| KI0810002 | 2008 | Oct | 2 |  | 2 |  |  |  |  |  |  |  |  |  |  |  |  |  |  | 2 | 0.001 |
| KI0810003 | 2008 | Oct | 4 |  | 15 | 2 | 21 |  |  |  |  |  |  |  |  |  |  |  |  | 38 | 0.850 |
| KI0810004 | 2008 | Oct |  |  |  |  |  |  |  |  |  |  |  |  |  |  |  |  |  | 0 | - |
| KI0810005 | 2008 | Oct | 25 |  |  |  | 1 | 1 |  |  |  |  |  |  |  |  |  |  |  | 2 | 0.693 |
| KI0810006 | 2008 | Oct | 25 |  |  |  | 1 | 1 |  |  |  |  |  | 1 |  |  |  |  |  | 3 | 1.099 |
| KI0810007 | 2008 | Oct | 2 |  | 13 |  | 1 |  |  | 2 |  |  |  |  |  |  |  |  |  | 16 | 0.602 |
| KI0810008 | 2008 | Oct | 5 |  | 11 |  |  | 14 |  |  | 1 |  |  |  |  |  |  |  |  | 26 | 0.823 |
| KI0810009 | 2008 | Oct |  |  |  |  |  |  |  |  |  |  |  |  |  |  |  |  |  | 0 | - |
| KI0810010 | 2008 | Oct | 4 |  | 29 |  | 39 |  |  |  | 1 |  |  |  | 4 |  |  |  |  | 73 | 0.920 |
| KI0810011 | 2008 | Oct | 17 |  | 4 |  |  |  |  |  |  |  |  |  | 46 |  |  |  |  | 50 | 0.279 |
| KI0810012 | 2008 | Oct | 2 |  | 7 | 5 | 4 |  |  |  |  |  |  |  |  |  |  |  | 1 | 17 | 1.232 |
| KI0810013 | 2008 | Oct | 4 |  |  |  | 15 | 2 |  |  | 1 |  |  |  |  |  |  |  |  | 18 | 0.557 |
| KI0810014 | 2008 | Oct | 25 |  |  |  | 2 | 2 |  |  |  |  |  |  |  |  |  |  |  | 4 | 0.693 |
| KI0810015 | 2008 | Oct | 4 |  | 5 |  | 10 | 1 |  |  |  |  |  | 1 |  |  |  |  |  | 17 | 1.005 |
| KI0810016 | 2008 | Oct | 1 | 63 |  |  | 48 |  | 3 |  |  |  |  |  |  |  |  |  |  | 114 | 0.788 |
| KI0810017 | 2008 | Oct | 17 |  | 2 |  | 7 | 20 |  | 1 |  |  |  |  | 60 |  |  |  |  | 90 | 0.938 |
| KI0810018 | 2008 | Oct | 1 | 91 |  |  | 31 | 2 |  |  | 10 |  |  |  | 1 |  |  |  |  | 135 | 0.958 |
| KI0810019 | 2008 | Oct | 2 | 2 | 21 | 2 |  | 4 | 2 |  | 3 |  |  |  | 19 |  |  |  |  | 53 | 1.463 |
| KI0810020 | 2008 | Oct |  |  |  |  |  |  |  |  |  |  |  |  |  |  |  |  |  | 0 | - |
| KI0810021 | 2008 | Oct | 4 |  | 1 |  | 2 |  |  |  |  |  |  |  | 1 |  |  |  |  | 4 | 1.040 |
| KI0810022 | 2008 | Oct | 4 |  | 5 |  | 27 |  |  |  |  |  |  | 1 |  |  |  |  |  | 33 | 0.556 |
| KI0810023 | 2008 | Oct | 4 | 4 | 9 |  | 29 |  |  |  |  |  |  | 1 | 4 |  |  |  |  | 47 | 1.194 |
| KI0810024 | 2008 | Oct | 4 |  | 7 |  | 54 |  |  |  |  |  |  |  |  |  |  |  |  | 61 | 0.356 |
| KI0810025 | 2008 | Oct | 25 |  | 1 |  |  |  |  |  |  |  |  |  |  |  |  |  | 1 | 2 | 0.693 |
| KI0810026 | 2008 | Oct | 17 | 6 | 5 | 4 | 1 | 14 |  | 2 |  |  |  |  | 52 |  |  |  |  | 84 | 1.320 |
| KI0810027 | 2008 | Oct |  |  |  |  |  |  |  |  |  |  |  |  |  |  |  |  |  | 0 | - |
| KI0810028 | 2008 | Oct | 17 |  | 2 |  |  |  |  |  |  |  |  | 1 | 15 |  |  |  |  | 18 | 0.557 |
| KI0810029 | 2008 | Oct | 2 |  | 9 |  |  |  |  |  |  |  |  |  | 8 |  | 6 |  |  | 23 | 1.085 |
| KI0810030 | 2008 | Oct | 4 |  | 2 |  | 56 |  |  |  | 1 |  |  |  |  |  |  |  |  | 59 | 0.233 |
| KI0810031 | 2008 | Oct |  |  |  |  |  |  |  |  |  |  |  |  |  |  |  |  |  | 0 | - |
| KI0810032 | 2008 | Oct | 17 |  | 8 |  |  | 2 |  |  |  |  |  |  | 59 |  |  |  |  | 69 | 0.486 |
| KI0810033 | 2008 | Oct |  |  |  |  |  |  |  |  |  |  |  |  |  |  |  |  |  | 0 | - |
| KI0810034 | 2008 | Oct | 2 |  | 3 |  |  |  |  |  | 2 |  |  |  | 2 |  |  |  | 1 | 8 | 1.321 |
| KI0810035 | 2008 | Oct | 17 |  |  | 3 |  | 1 |  |  |  |  |  | 1 | 31 |  |  |  |  | 36 | 0.535 |
| KI0810036 | 2008 | Oct | 2 |  | 6 |  | 2 |  |  |  |  |  |  |  |  |  |  |  |  | 8 | 0.562 |
| KI0810037 | 2008 | Oct | 9 |  | 2 |  | 2 |  |  |  | 3 |  |  |  |  |  |  |  |  | 7 | 1.079 |
| KI0810038 | 2008 | Oct | 2 |  | 5 |  |  |  |  |  |  |  |  |  |  |  |  |  |  | 5 | 0.001 |
| KI0810039 | 2008 | Oct | 17 |  | 5 |  | 4 | 35 |  |  | 2 |  |  |  | 96 |  |  |  |  | 142 | 0.888 |
| KI0810040 | 2008 | Oct |  |  |  |  |  |  |  |  |  |  |  |  |  |  |  |  |  | 0 | - |
| KI0810041 | 2008 | Oct | 17 |  | 1 |  | 7 | 24 |  |  |  |  |  |  | 56 |  |  |  |  | 88 | 0.894 |
| KI0411001 | 2004 | Nov | 13 |  |  |  |  |  |  |  |  | 21 |  |  |  |  |  |  |  | 21 | 0.001 |
| KI0411002 | 2004 | Nov | 3 |  |  | 57 |  |  |  |  |  | 37 |  |  |  |  |  |  |  | 94 | 0.670 |
| KI0411003 | 2004 | Nov | 3 |  |  | 114 | 1 |  |  |  |  |  |  |  |  |  |  |  |  | 115 | 0.050 |
| KI0411004 | 2004 | Nov | 4 |  |  | 11 | 39 |  |  |  |  |  |  |  |  |  |  |  |  | 50 | 0.527 |
| KI0411005 | 2004 | Nov | 4 |  |  | 22 | 32 |  |  |  |  |  |  |  |  |  |  |  |  | 54 | 0.676 |
| KI0411006 | 2004 | Nov | 3 |  |  | 212 |  |  |  |  |  |  |  |  |  |  |  |  |  | 212 | 0.001 |
| KI0411007 | 2004 | Nov | 3 |  |  | 853 | 31 |  |  |  |  |  |  |  |  |  |  |  |  | 884 | 0.161 |
| KI0411008 | 2004 | Nov | 3 |  |  | 4 |  |  |  |  |  |  |  |  |  |  |  |  |  | 4 | 0.001 |
| KI0411009 | 2004 | Nov | 3 |  |  | 20 | 7 |  |  |  |  | 2 |  |  |  |  |  |  |  | 29 | 0.784 |
| KI0411010 | 2004 | Nov | 3 |  |  | 15 |  |  |  |  |  | 1 |  |  |  |  |  |  |  | 16 | 0.777 |
| KI0411011 | 2004 | Nov | 3 |  |  | 124 |  |  |  |  |  | 1 |  |  |  |  |  |  |  | 125 | 0.047 |
| KI0411012 | 2004 | Nov | 3 |  |  | 114 |  |  |  |  |  |  |  |  |  |  |  |  |  | 114 | 0.001 |
| KI0411013 | 2004 | Nov | 3 |  |  | 834 | 4 |  | 1 |  | 1 |  |  |  |  |  |  |  |  | 840 | 0.049 |
| KI0411014 | 2004 | Nov |  |  |  |  |  |  |  |  |  |  |  |  |  |  |  |  |  | 0 | - |
| KI0411015 | 2004 | Nov | 3 |  |  | 5 |  |  |  |  |  |  | 1 |  |  |  |  |  |  | 6 | 0.451 |
| KI0411016 | 2004 | Nov |  |  |  |  |  |  |  |  |  |  |  |  |  |  |  |  |  | 0 | - |
| KI0411017 | 2004 | Nov | 3 |  |  | 72 |  |  |  |  |  |  |  |  |  |  |  |  |  | 72 | 0.001 |
| KI0411018 | 2004 | Nov | 3 |  |  | 41 | 23 |  |  |  |  |  |  |  |  |  |  |  |  | 64 | 0.653 |
| KI0411019 | 2004 | Nov | 3 | 1 |  | 478 | 51 |  |  |  |  |  |  |  |  |  |  |  |  | 530 | 0.330 |
| KI0411020 | 2004 | Nov |  |  |  |  |  |  |  |  |  |  |  |  |  |  |  |  |  | 0 | - |
| KI0411021 | 2004 | Nov | 13 |  |  | 1 |  |  |  |  |  | 30 | 3 |  |  |  |  |  |  | 34 | 0.428 |
| KI0411022 | 2004 | Nov | 3 |  |  | 645 | 1 |  | 1 |  |  |  |  |  |  |  |  |  |  | 647 | 0.023 |
| KI0411023 | 2004 | Nov | 3 |  |  | 40 | 5 |  |  |  |  | 1 |  |  |  |  |  |  |  | 46 | 0.539 |
| KI0411024 | 2004 | Nov | 3 |  |  | 20 |  |  |  |  |  |  | 3 |  |  |  |  |  |  | 23 | 0.387 |
| KI0411025 | 2004 | Nov | 3 |  |  | 16 |  |  |  |  |  |  |  |  |  |  |  |  |  | 16 | 0.001 |
| KI0411026 | 2004 | Nov | 3 |  |  | 66 | 6 |  |  |  |  |  |  |  |  |  |  |  |  | 72 | 0.509 |
| KI0411027 | 2004 | Nov |  |  |  |  |  |  |  |  |  |  |  |  |  |  |  |  |  | 0 | - |
| KI0411028 | 2004 | Nov | 13 |  |  | 15 |  |  |  |  |  | 21 |  |  |  |  |  |  |  | 36 | 0.992 |
| KI0411029 | 2004 | Nov | 3 |  |  | 47 |  |  |  |  |  | 2 | 5 |  |  |  |  |  |  | 54 | 0.546 |
| KI0411030 | 2004 | Nov |  |  |  |  |  |  |  |  |  |  |  |  |  |  |  |  |  | 0 | - |
| KI0411031 | 2004 | Nov | 3 |  |  | 15 |  |  |  |  |  | 5 | 4 |  |  |  |  |  |  | 24 | 0.919 |
| KI0411032 | 2004 | Nov | 3 |  |  | 21 |  |  |  |  |  | 4 | 1 |  |  |  |  |  |  | 26 | 0.586 |
| KI0411033 | 2004 | Nov | 3 |  |  | 26 |  |  |  |  |  |  | 9 |  |  |  |  |  | 232 | 267 | 0.556 |
| KI0411034 | 2004 | Nov | 3 |  |  | 110 |  |  |  |  |  | 4 | 1 |  |  |  |  |  |  | 115 | 0.201 |
| KI0411035 | 2004 | Nov |  |  |  |  |  |  |  |  |  |  |  |  |  |  |  |  |  | 0 | - |
| KI0411036 | 2004 | Nov | 3 |  |  | 26 | 1 |  |  |  |  | 14 | 16 |  |  |  |  |  |  | 57 | 1.130 |
| KI0411037 | 2004 | Nov |  |  |  |  |  |  |  |  |  |  |  |  |  |  |  |  |  | 0 | - |
| KI0411038 | 2004 | Nov | 13 |  |  | 2 |  |  |  |  |  | 20 | 1 |  |  |  |  |  |  | 23 | 0.923 |
| KI0411039 | 2004 | Nov |  |  |  |  |  |  |  |  |  |  |  |  |  |  |  |  |  | 0 | - |
| KI0411040 | 2004 | Nov | 4 |  |  | 19 | 29 |  |  |  |  | 2 |  |  |  |  |  |  |  | 50 | 0.812 |
| KI0411041 | 2004 | Nov | 3 |  |  | 30 |  |  |  |  |  |  |  |  |  |  |  |  |  | 30 | 0.625 |
| KI0411042 | 2004 | Nov | 3 |  |  | 7 |  |  |  |  |  | 1 | 3 |  |  |  |  |  |  | 11 | 1.075 |
| KI0411043 | 2004 | Nov | 14 |  |  | 24 |  |  |  |  |  |  | 25 |  |  |  |  |  |  | 49 | 0.777 |
| KI0411044 | 2004 | Nov |  |  |  |  |  |  |  |  |  |  |  |  |  |  |  |  |  | 0 | - |
| KI0411045 | 2004 | Nov | 3 |  |  | 15 | 13 | 1 |  |  |  |  |  |  |  |  |  |  |  | 29 | 0.817 |
| KI0511001 | 2005 | Nov | 1 | 370 | 3 | 10 | 153 |  |  |  |  |  |  |  |  |  |  |  |  | 536 | 0.717 |
| KI0511002 | 2005 | Nov | 4 |  |  |  | 62 |  |  |  |  |  |  |  |  |  |  |  |  | 62 | 0.001 |
| KI0511003 | 2005 | Nov | 4 |  |  |  | 10 |  |  |  |  |  |  |  |  |  |  |  |  | 10 | 0.001 |
| KI0511004 | 2005 | Nov | 4 |  |  |  | 8 |  |  |  |  |  |  |  |  |  |  |  |  | 8 | 0.001 |
| KI0511005 | 2005 | Nov | 4 |  |  |  | 93 |  |  |  |  |  |  |  |  |  |  |  |  | 93 | 0.001 |
| KI0511006 | 2005 | Nov | 4 |  |  | 3 | 51 |  |  |  |  |  |  |  |  |  |  |  |  | 54 | 0.215 |
| KI0511007 | 2005 | Nov | 4 |  |  |  | 102 |  |  |  |  |  |  |  |  |  |  |  |  | 102 | 0.001 |
| KI0511008 | 2005 | Nov | 4 |  |  |  | 139 |  |  |  |  |  |  |  |  |  |  |  |  | 139 | 0.001 |
| KI0511009 | 2005 | Nov |  |  |  | 1 | 1 |  |  |  |  |  |  |  |  |  |  |  |  | 2 | 0.693 |
| KI0511010 | 2005 | Nov | 4 |  |  |  | 23 | 8 |  |  |  |  |  |  |  |  |  |  |  | 31 | 0.571 |
| KI0511011 | 2005 | Nov | 4 |  |  |  | 6 |  | 1 |  |  |  |  |  | 2 |  |  |  |  | 9 | 1.089 |
| KI0511012 | 2005 | Nov | 4 |  |  | 164 | 433 |  |  |  |  |  |  |  |  |  |  |  |  | 597 | 0.588 |
| KI0511013 | 2005 | Nov | 4 |  | 4 | 29 | 251 |  |  |  |  |  |  |  |  |  |  |  |  | 284 | 0.402 |
| KI0511014 | 2005 | Nov | 4 |  |  |  | 34 |  | 6 |  |  |  |  |  |  |  |  |  |  | 40 | 0.423 |
| KI0511015 | 2005 | Nov | 4 |  |  |  | 5 |  |  |  |  |  |  |  |  |  |  |  |  | 5 | 0.001 |
| KI0711001 | 2007 | Nov | 4 |  |  | 1 | 258 |  | 2 |  |  |  |  |  |  |  |  |  |  | 261 | 0.070 |
| KI0711002 | 2007 | Nov | 4 |  |  | 33 | 515 |  | 2 | 2 |  |  |  |  |  |  |  |  |  | 552 | 0.274 |
| KI0711003 | 2007 | Nov |  |  |  |  |  |  |  |  |  |  |  |  |  |  |  |  |  | 0 | - |
| KI0711004 | 2007 | Nov | 4 | 2 |  |  | 78 |  |  |  |  |  |  |  |  |  |  |  |  | 80 | 0.117 |
| KI0711005 | 2007 | Nov | 4 | 10 |  | 6 | 65 |  |  |  |  |  |  |  |  |  |  |  |  | 81 | 0.628 |
| KI0711006 | 2007 | Nov | 3 |  |  | 138 | 91 |  |  |  | 1 |  |  |  |  |  |  |  |  | 230 | 0.697 |
| KI0711007 | 2007 | Nov | 4 |  |  | 25 | 68 | 1 |  |  |  |  |  |  |  |  |  |  |  | 94 | 0.635 |
| KI0711008 | 2007 | Nov | 4 | 104 |  | 71 | 347 |  |  | 4 |  |  |  |  |  |  |  |  |  | 526 | 0.902 |
| KI0711009 | 2007 | Nov |  |  |  |  |  |  |  |  |  |  |  |  |  |  |  |  |  | 0 | - |
| KI0711010 | 2007 | Nov | 3 |  |  | 155 | 103 |  |  | 1 |  |  |  |  |  |  |  |  |  | 259 | 0.735 |
| KI0711011 | 2007 | Nov |  |  |  |  |  |  |  |  |  |  |  |  |  |  |  |  |  | 0 | - |
| KI0711012 | 2007 | Nov | 3 |  |  | 108 | 60 |  |  | 1 | 2 |  |  |  |  |  |  |  |  | 171 | 0.740 |
| KI0711013 | 2007 | Nov | 3 | 2 |  | 427 | 316 |  |  |  |  |  |  |  |  |  |  |  |  | 745 | 0.708 |
| KI0711014 | 2007 | Nov |  |  |  |  |  |  |  |  |  |  |  |  |  |  |  |  |  | 0 | - |
| KI0711015 | 2007 | Nov |  |  |  |  |  |  |  |  |  |  |  |  |  |  |  |  |  | 0 | - |
| KI0711016 | 2007 | Nov |  |  |  |  |  |  |  |  |  |  |  |  |  |  |  |  |  | 0 | - |
| KI0711017 | 2007 | Nov |  |  |  |  |  |  |  |  |  |  |  |  |  |  |  |  |  | 0 | - |
| KI0711018 | 2007 | Nov |  |  |  |  |  |  |  |  |  |  |  |  |  |  |  |  |  | 0 | - |
| KI0711019 | 2007 | Nov | 4 |  |  | 25 | 95 |  |  |  |  |  |  |  |  |  |  |  |  | 120 | 0.512 |
| KI0711020 | 2007 | Nov | 4 |  |  | 3 | 297 |  |  |  | 1 |  |  |  |  |  |  |  |  | 301 | 0.078 |
| KI0711021 | 2007 | Nov |  |  |  |  |  |  |  |  |  |  |  |  |  |  |  |  |  | 0 | - |
| KI0711022 | 2007 | Nov | 4 |  |  | 1 | 259 |  |  |  |  |  |  |  |  |  |  |  |  | 260 | 0.050 |
| KI0711023 | 2007 | Nov |  |  |  |  |  |  |  |  |  |  |  |  |  |  |  |  |  | 0 | - |
| KI0811001 | 2008 | Nov | 4 |  |  | 1 | 68 |  |  |  |  |  |  |  |  |  |  |  |  | 69 | 0.076 |
| KI0811002 | 2008 | Nov | 4 |  | 1 | 4 | 41 |  |  |  |  |  |  |  |  |  |  |  |  | 46 | 0.398 |
| KI0811003 | 2008 | Nov | 3 |  | 25 | 82 | 6 | 18 |  |  | 1 |  |  |  |  |  |  |  |  | 132 | 1.060 |
| KI0811004 | 2008 | Nov | 1 | 86 | 41 |  | 58 |  |  |  |  |  |  |  |  |  |  |  |  | 185 | 1.172 |
| KI0811005 | 2008 | Nov | 3 | 28 | 6 | 184 | 102 | 15 |  | 1 |  |  |  |  |  |  |  |  |  | 336 | 1.250 |
| KI0811006 | 2008 | Nov | 3 | 2 | 7 | 126 | 24 | 31 |  | 1 |  |  |  |  |  |  |  |  |  | 191 | 1.027 |
| KI0811007 | 2008 | Nov | 5 |  |  | 1 | 18 | 27 |  |  |  |  |  |  |  |  |  |  |  | 46 | 0.763 |
| KI0811008 | 2008 | Nov | 3 |  |  | 207 | 34 | 4 |  | 1 |  |  |  |  |  |  |  |  |  | 246 | 0.567 |
| KI0811009 | 2008 | Nov | 3 |  | 1 | 98 | 10 | 24 |  |  |  |  |  |  |  |  |  |  |  | 133 | 0.765 |
| KI0811010 | 2008 | Nov | 4 | 3 | 10 |  | 70 | 25 |  |  |  |  |  |  |  |  |  |  |  | 108 | 1.203 |
| KI0811011 | 2008 | Nov | 3 | 3 | 12 | 466 | 24 | 13 |  |  |  |  |  |  |  |  |  |  |  | 518 | 0.563 |
| KI0811012 | 2008 | Nov | 4 |  | 8 | 16 | 56 | 22 |  |  |  |  |  |  |  |  |  |  |  | 102 | 1.358 |
| KI0811013 | 2008 | Nov | 3 |  |  | 80 |  | 19 |  |  |  |  |  |  |  |  |  |  |  | 99 | 0.489 |
| KI0811014 | 2008 | Nov | 4 |  |  | 2 | 8 |  |  |  |  |  |  |  |  |  |  |  |  | 10 | 0.500 |
| KI0811015 | 2008 | Nov | 3 |  | 3 | 248 | 6 |  |  |  |  |  |  |  |  |  |  |  |  | 257 | 0.174 |
| KI0811016 | 2008 | Nov | 4 |  |  |  | 53 | 20 |  | 1 |  |  |  |  |  |  |  |  |  | 74 | 0.713 |
| KI0811017 | 2008 | Nov | 4 |  | 2 |  | 7 |  |  |  |  |  |  |  |  |  |  |  |  | 9 | 0.530 |
| KI0811018 | 2008 | Nov | 3 |  |  | 319 | 146 |  |  | 1 |  |  |  |  |  |  |  |  |  | 466 | 0.661 |
| KI0811019 | 2008 | Nov | 3 |  |  | 187 | 69 |  |  |  |  |  |  |  |  |  |  |  |  | 256 | 0.583 |
| KI0811020 | 2008 | Nov | 4 |  |  | 14 | 374 |  |  |  |  |  |  |  |  |  |  |  |  | 388 | 0.190 |
| KI0811021 | 2008 | Nov | 3 |  |  | 320 | 68 | 1 |  |  |  |  |  |  |  |  |  |  |  | 389 | 0.497 |
| KI0811022 | 2008 | Nov | 4 |  |  | 64 | 294 |  |  |  |  |  |  |  |  |  |  |  |  | 358 | 0.470 |
| KI0811023 | 2008 | Nov | 4 |  | 1 | 14 | 252 |  |  |  |  |  |  |  |  |  |  |  |  | 267 | 0.230 |
| KI0811024 | 2008 | Nov | 4 |  | 1 | 2 | 164 |  |  | 2 |  |  |  |  |  |  |  |  |  | 169 | 0.200 |
| KI0811025 | 2008 | Nov | 3 |  | 5 | 243 | 3 |  |  |  |  |  |  |  |  |  |  |  |  | 251 | 0.207 |
| KI0811026 | 2008 | Nov | 4 |  |  | 4 | 191 |  |  |  |  |  |  |  |  |  |  |  |  | 195 | 0.100 |
| KI0811027 | 2008 | Nov | 4 |  |  | 57 | 218 |  |  |  |  |  |  |  |  |  |  |  |  | 275 | 0.564 |
| KI0811028 | 2008 | Nov | 4 |  |  | 2 | 45 |  |  |  |  |  |  |  |  |  |  |  |  | 47 | 0.274 |
| KI0811029 | 2008 | Nov | 4 |  |  | 152 | 497 |  |  |  |  |  |  |  | 6 |  |  |  |  | 655 | 0.591 |
| KI0811030 | 2008 | Nov | 4 | 1 |  | 55 | 201 |  |  |  |  |  |  |  |  |  |  |  |  | 257 | 0.585 |
| KI0811031 | 2008 | Nov | 4 |  |  | 25 | 281 | 2 |  |  |  |  |  |  |  |  |  |  |  | 308 | 0.341 |
| KI0811032 | 2008 | Nov | 4 |  | 1 |  | 88 | 19 |  |  |  |  |  |  |  |  |  |  |  | 108 | 0.563 |
| KI0811033 | 2008 | Nov | 4 |  |  | 8 | 151 |  |  |  |  |  |  |  |  |  |  |  |  | 159 | 0.199 |
| KI0811034 | 2008 | Nov | 4 |  |  | 4 | 237 |  |  |  |  |  |  |  |  |  |  |  |  | 241 | 0.084 |
| KI0811035 | 2008 | Nov | 3 |  |  | 87 | 79 |  |  |  |  |  |  |  |  |  |  |  |  | 166 | 0.692 |
| KI0811036 | 2008 | Nov | 4 |  |  | 50 | 148 | 16 |  |  |  |  |  |  |  |  |  |  |  | 214 | 0.789 |
| KI0811037 | 2008 | Nov | 4 |  |  | 3 | 20 | 5 |  |  |  |  |  |  |  |  |  |  |  | 28 | 0.787 |
| KI0811038 | 2008 | Nov | 4 |  |  | 64 | 133 |  |  |  |  |  |  |  |  |  |  |  |  | 197 | 0.630 |
| KI0811039 | 2008 | Nov | 4 |  |  | 90 | 204 |  |  |  |  |  |  |  |  |  |  |  |  | 294 | 0.616 |
| Total | |  |  | 1926 | 1047 | 9193 | 12235 | 603 | 1353 | 128 | 205 | 166 | 98 | 783 | 2056 | 247 | 837 | 54 | 235 | 31166 |  |

b.

| Sample | Year | Month | *DPS* | Species | | | | | | | | | | | | | | | | Total | *IR* |
| --- | --- | --- | --- | --- | --- | --- | --- | --- | --- | --- | --- | --- | --- | --- | --- | --- | --- | --- | --- | --- | --- |
|  |  |  |  | 1 | 2 | 3 | 4 | 5 | 6 | 7 | 9 | 13 | 14 | 16 | 17 | 18 | 19 | 21 | X |  |  |
| KI0409001 | 2004 | Sep | 14 |  |  |  |  |  |  |  |  |  | 1 |  |  |  |  |  |  | 1 | 1.000 |
| KI0409002 | 2004 | Sep | 2 |  | 2 |  |  |  |  |  |  |  |  |  |  |  |  |  |  | 2 | 0.286 |
| KI0409003 | 2004 | Sep | 2 |  | 0 |  |  |  |  |  |  |  |  |  |  |  |  |  |  | 0 | 0.000 |
| KI0509001 | 2005 | Sep | 16 |  |  |  |  |  |  |  |  |  |  | 62 | 29 |  | 22 |  |  | 113 | 1.000 |
| KI0509002 | 2005 | Sep |  |  |  |  |  |  |  |  |  |  |  |  |  |  |  |  |  |  |  |
| KI0509003 | 2005 | Sep | 18 |  |  |  |  |  |  |  |  |  |  | 30 | 25 | 60 |  |  |  | 115 | 0.991 |
| KI0509004 | 2005 | Sep | 16 |  |  |  |  |  |  |  |  |  |  | 1 |  |  |  |  |  | 1 | 1.000 |
| KI0509005 | 2005 | Sep | 16 |  |  |  |  |  |  |  |  |  |  | 5 |  |  |  |  |  | 5 | 1.000 |
| KI0509006 | 2005 | Sep | 16 |  |  |  |  |  | 2 |  |  |  |  | 49 | 14 |  |  |  |  | 65 | 0.985 |
| KI0509007 | 2005 | Sep | 18 |  |  |  |  |  |  |  |  |  |  | 35 | 26 | 50 |  |  |  | 111 | 0.982 |
| KI0509008 | 2005 | Sep |  |  |  |  |  |  |  |  |  |  |  |  |  |  |  |  |  |  |  |
| KI0509009 | 2005 | Sep | 16 |  |  |  |  |  |  |  |  |  |  | 1 |  |  |  |  |  | 1 | 1.000 |
| KI0509010 | 2005 | Sep | 16 |  |  |  |  |  |  |  |  |  |  | 11 |  |  |  |  |  | 11 | 0.407 |
| KI0509011 | 2005 | Sep | 16 |  |  |  |  |  |  |  |  |  |  | 3 |  |  |  |  |  | 3 | 0.750 |
| KI0709001 | 2007 | Sep | 17 |  |  |  |  |  |  |  |  |  |  |  | 4 |  |  |  |  | 4 | 1.000 |
| KI0709002 | 2007 | Sep | 17 |  |  |  |  |  |  |  |  |  |  | 0 | 62 |  | 5 |  |  | 67 | 0.985 |
| KI0709003 | 2007 | Sep | 18 |  |  |  |  |  |  |  |  |  |  |  |  | 11 |  |  |  | 11 | 0.196 |
| KI0709004 | 2007 | Sep | 17 |  |  |  |  |  |  | 1 |  |  |  |  | 18 |  | 7 |  |  | 27 | 1.000 |
| KI0709005 | 2007 | Sep | 17 |  |  |  |  |  |  | 1 |  |  |  |  | 6 |  | 2 |  |  | 9 | 0.900 |
| KI0709006 | 2007 | Sep | 7 |  |  |  |  |  |  | 1 |  |  |  |  |  |  | 26 |  |  | 27 | 0.491 |
| KI0709007 | 2007 | Sep |  |  |  |  |  |  |  |  |  |  |  |  |  |  |  |  |  |  |  |
| KI0709008 | 2007 | Sep | 19 |  |  |  |  |  |  |  |  |  |  |  |  |  | 44 |  |  | 44 | 1.000 |
| KI0709009 | 2007 | Sep | 7 |  |  |  |  |  |  | 4 |  |  |  |  | 3 |  |  |  |  | 7 | 1.000 |
| KI0709010 | 2007 | Sep | 17 |  |  |  |  |  |  | 0 |  |  |  |  | 77 |  |  |  | 1 | 78 | 0.975 |
| KI0709011 | 2007 | Sep | 7 |  |  |  |  |  |  | 0 |  |  |  |  | 5 |  | 8 |  |  | 13 | 0.500 |
| KI0709012 | 2007 | Sep | 19 |  |  |  |  |  |  | 0 |  |  |  |  |  |  | 19 |  |  | 19 | 0.864 |
| KI0709013 | 2007 | Sep | 7 |  |  |  |  |  |  | 0 |  |  |  |  |  |  | 2 |  |  | 2 | 0.400 |
| KI0709014 | 2007 | Sep | 5 |  |  |  |  |  |  | 0 |  |  |  |  | 18 |  | 1 |  |  | 19 | 0.333 |
| KI0709015 | 2007 | Sep | 19 |  |  |  |  |  |  |  |  |  |  |  |  |  | 51 |  |  | 51 | 1.000 |
| KI0709016 | 2007 | Sep | 17 |  |  |  |  |  |  |  |  |  |  |  | 26 |  | 8 |  |  | 34 | 1.000 |
| KI0709017 | 2007 | Sep | 17 |  |  |  |  |  |  | 1 |  |  |  |  | 65 |  | 18 |  |  | 84 | 1.000 |
| KI0709018 | 2007 | Sep | 17 |  |  |  |  |  |  |  |  |  |  |  | 140 |  | 5 |  |  | 145 | 1.000 |
| KI0709019 | 2007 | Sep |  |  |  |  |  |  |  |  |  |  |  |  |  |  |  |  |  |  |  |
| KI0709020 | 2007 | Sep | 17 |  |  |  |  |  |  | 0 |  |  |  |  | 10 |  |  |  |  | 10 | 0.833 |
| KI0709021 | 2007 | Sep | 1 | 157 | 1 |  |  |  |  | 0 |  |  |  | 1 |  |  | 98 |  |  | 257 | 0.996 |
| KI0709022 | 2007 | Sep |  |  |  |  |  |  |  |  |  |  |  |  |  |  |  |  |  |  |  |
| KI0709023 | 2007 | Sep | 19 |  | 1 |  |  |  |  |  |  |  |  |  |  |  | 26 |  |  | 27 | 0.964 |
| KI0709024 | 2007 | Sep | 19 |  |  |  |  |  |  |  |  |  |  |  | 2 |  | 6 |  |  | 8 | 1.000 |
| KI0709025 | 2007 | Sep | 17 |  |  |  |  |  |  | 1 |  |  |  |  | 6 |  | 3 |  |  | 10 | 1.000 |
| KI0709026 | 2007 | Sep | 19 |  |  |  |  |  |  |  |  |  |  |  | 3 |  | 11 |  |  | 14 | 1.000 |
| KI0709027 | 2007 | Sep | 19 |  |  |  |  |  |  | 1 |  |  |  |  |  |  | 134 |  |  | 135 | 1.000 |
| KI0709028 | 2007 | Sep | 19 |  |  |  |  |  |  | 0 |  |  |  |  | 6 |  | 29 |  |  | 35 | 0.972 |
| KI0709029 | 2007 | Sep | 17 |  |  |  |  |  |  | 2 |  |  |  |  | 29 |  | 16 |  |  | 47 | 1.000 |
| KI0809001 | 2008 | Sep | 17 |  |  |  |  |  |  |  |  |  |  |  | 89 |  |  |  |  | 89 | 1.000 |
| KI0809002 | 2008 | Sep | 17 |  |  |  |  |  |  |  |  |  |  | 4 | 61 |  |  |  |  | 65 | 1.000 |
| KI0809003 | 2008 | Sep | 17 |  | 0 |  |  |  |  |  |  |  |  | 1 | 42 |  |  |  |  | 43 | 0.896 |
| KI0809004 | 2008 | Sep | 16 |  | 0 |  |  |  |  |  |  |  |  | 3 | 7 | 2 |  |  |  | 12 | 0.231 |
| KI0809005 | 2008 | Sep | 17 |  |  |  |  |  |  |  |  |  |  | 12 | 41 |  |  |  |  | 53 | 0.981 |
| KI0809006 | 2008 | Sep | 17 |  |  |  |  |  |  |  |  |  |  | 0 | 8 |  |  |  |  | 8 | 0.889 |
| KI0809007 | 2008 | Sep | 16 |  | 2 |  |  |  |  |  |  |  |  | 10 | 3 |  |  |  |  | 15 | 0.395 |
| KI0809008 | 2008 | Sep | 17 |  |  |  |  |  |  |  |  |  |  |  | 44 |  | 1 |  |  | 45 | 1.000 |
| KI0809009 | 2008 | Sep | 16 |  |  |  |  |  |  | 1 |  |  |  | 16 | 2 | 1 |  |  |  | 20 | 0.645 |
| KI0809010 | 2008 | Sep | 16 |  | 1 |  |  |  |  |  |  |  |  | 32 | 19 |  |  |  |  | 52 | 0.929 |
| KI0809011 | 2008 | Sep | 16 |  |  |  |  |  |  |  |  |  |  | 29 |  |  |  |  |  | 29 | 0.354 |
| KI0809012 | 2008 | Sep | 2 |  | 2 |  |  |  |  |  |  |  |  | 158 | 48 |  |  |  |  | 208 | 0.967 |
| KI0809013 | 2008 | Sep | 17 |  |  |  |  |  |  |  |  |  |  | 6 | 14 |  |  |  |  | 20 | 1.000 |
| KI0809014 | 2008 | Sep | 16 |  | 1 |  |  |  |  |  |  |  |  | 3 | 15 |  |  |  |  | 19 | 0.260 |
| KI0809015 | 2008 | Sep | 17 |  |  |  |  |  |  |  |  |  |  | 2 | 62 |  |  |  |  | 64 | 0.889 |
| KI0809016 | 2008 | Sep | 17 |  |  |  |  |  |  | 2 |  |  |  | 4 | 44 |  |  |  |  | 50 | 0.877 |
| KI0809017 | 2008 | Sep | 17 |  |  |  |  |  |  |  |  |  |  | 8 | 89 |  | 2 |  |  | 99 | 0.811 |
| KI0809018 | 2008 | Sep | 17 |  | 0 |  |  |  |  |  |  |  |  |  | 103 |  | 1 |  |  | 104 | 0.981 |
| KI0809019 | 2008 | Sep |  |  |  |  |  |  |  |  |  |  |  |  |  |  |  |  |  |  |  |
| KI0809020 | 2008 | Sep |  |  |  |  |  |  |  |  |  |  |  |  |  |  |  |  |  |  |  |
| KI0809021 | 2008 | Sep |  |  |  |  |  |  |  |  |  |  |  |  |  |  |  |  |  |  |  |
| KI0809022 | 2008 | Sep |  |  |  |  |  |  |  |  |  |  |  |  |  |  |  |  |  |  |  |
| KI0809023 | 2008 | Sep |  |  |  |  |  |  |  |  |  |  |  |  |  |  |  |  |  |  |  |
| KI0809024 | 2008 | Sep |  |  |  |  |  |  |  |  |  |  |  |  |  |  |  |  |  |  |  |
| KI0809025 | 2008 | Sep |  |  |  |  |  |  |  |  |  |  |  |  |  |  |  |  |  |  |  |
| KI0809026 | 2008 | Sep |  |  |  |  |  |  |  |  |  |  |  |  |  |  |  |  |  |  |  |
| KI0809027 | 2008 | Sep |  |  |  |  |  |  |  |  |  |  |  |  |  |  |  |  |  |  |  |
| KI0809028 | 2008 | Sep |  |  |  |  |  |  |  |  |  |  |  |  |  |  |  |  |  |  |  |
| KI0809029 | 2008 | Sep |  |  |  |  |  |  |  |  |  |  |  |  |  |  |  |  |  |  |  |
| KI0809030 | 2008 | Sep |  |  |  |  |  |  |  |  |  |  |  |  |  |  |  |  |  |  |  |
| KI0809031 | 2008 | Sep |  |  |  |  |  |  |  |  |  |  |  |  |  |  |  |  |  |  |  |
| KI0809032 | 2008 | Sep |  |  |  |  |  |  |  |  |  |  |  |  |  |  |  |  |  |  |  |
| KI0809033 | 2008 | Sep |  |  |  |  |  |  |  |  |  |  |  |  |  |  |  |  |  |  |  |
| KI0809034 | 2008 | Sep |  |  |  |  |  |  |  |  |  |  |  |  |  |  |  |  |  |  |  |
| KI0809035 | 2008 | Sep |  |  |  |  |  |  |  |  |  |  |  |  |  |  |  |  |  |  |  |
| KI0809036 | 2008 | Sep |  |  |  |  |  |  |  |  |  |  |  |  |  |  |  |  |  |  |  |
| KI0010001 | 2000 | Oct | 1 | 4 |  |  |  |  |  |  |  |  |  |  |  |  |  |  |  | 4 | 1.000 |
| KI0010002 | 2000 | Oct | 2 |  | 0 |  |  |  |  |  |  |  |  |  |  |  |  |  |  | 0 | 0.000 |
| KI0010003 | 2000 | Oct | 2 |  | 0 |  |  |  |  |  |  |  |  |  |  |  |  |  |  | 0 | 0.000 |
| KI0010004 | 2000 | Oct |  |  |  |  |  |  |  |  |  |  |  |  |  |  |  |  |  |  |  |
| KI0010005 | 2000 | Oct | 25 |  |  |  |  | 1 |  |  |  |  |  |  |  |  |  |  |  | 1 | 0.500 |
| KI0010006 | 2000 | Oct | 2 |  | 1 |  |  |  |  |  |  |  |  |  |  |  |  |  |  | 1 | 1.000 |
| KI0010007 | 2000 | Oct | 2 |  | 1 |  |  |  |  |  |  |  |  |  |  |  |  |  |  | 1 | 1.000 |
| KI0010008 | 2000 | Oct | 2 |  | 2 |  |  |  |  |  |  |  |  |  |  |  |  |  |  | 2 | 1.000 |
| KI0010009 | 2000 | Oct |  |  |  |  |  |  |  |  |  |  |  |  |  |  |  |  |  |  |  |
| KI0010010 | 2000 | Oct |  |  |  |  |  |  |  |  |  |  |  |  |  |  |  |  |  |  |  |
| KI0010011 | 2000 | Oct | 2 | 2 | 1 |  | 1 |  | 0 |  |  |  |  |  |  |  |  |  |  | 4 | 0.571 |
| KI0010012 | 2000 | Oct | 2 |  | 23 |  |  |  |  |  |  |  |  |  |  |  |  |  |  | 23 | 1.000 |
| KI0010013 | 2000 | Oct | 6 |  |  |  |  |  | 4 |  |  |  |  |  |  |  |  |  |  | 4 | 0.071 |
| KI0010014 | 2000 | Oct | 2 |  | 0 | 1 |  |  | 1 |  |  |  |  |  |  |  |  |  |  | 2 | 0.051 |
| KI0010015 | 2000 | Oct | 1 | 49 | 1 |  |  |  | 7 |  |  |  |  |  |  |  |  |  |  | 57 | 0.713 |
| KI0010016 | 2000 | Oct | 2 | 1 | 0 |  |  |  |  |  |  |  |  |  |  |  |  |  |  | 2 | 0.105 |
| KI0010017 | 2000 | Oct | 6 |  |  |  |  |  | 2 |  |  |  |  |  |  |  |  |  |  | 2 | 0.500 |
| KI0010018 | 2000 | Oct | 3 | 1 | 0 | 10 |  |  |  |  |  |  |  |  |  |  |  |  |  | 11 | 0.647 |
| KI0010019 | 2000 | Oct | 2 | 8 | 0 |  |  | 8 | 6 |  |  |  |  |  |  |  |  |  |  | 22 | 0.247 |
| KI0010020 | 2000 | Oct | 3 |  |  | 9 |  |  |  |  |  |  |  |  |  |  |  |  |  | 9 | 1.000 |
| KI0010021 | 2000 | Oct | 2 |  | 0 |  |  |  | 4 |  |  |  |  |  |  |  |  |  |  | 4 | 0.190 |
| KI0010022 | 2000 | Oct | 1 | 106 | 2 | 5 |  |  | 1 | 0 |  |  |  |  |  |  |  |  |  | 114 | 0.699 |
| KI0010023 | 2000 | Oct |  |  |  |  |  |  |  |  |  |  |  |  |  |  |  |  |  |  |  |
| KI0010024 | 2000 | Oct | 5 |  |  |  |  | 5 |  |  |  |  |  |  |  |  |  |  |  | 6 | 1.000 |
| KI0010025 | 2000 | Oct | 2 |  | 1 |  |  | 10 |  |  |  |  | 1 |  |  |  |  |  |  | 12 | 0.375 |
| KI0010026 | 2000 | Oct | 2 |  | 2 |  |  | 5 | 1 |  |  |  |  |  |  |  |  |  |  | 8 | 0.348 |
| KI0010027 | 2000 | Oct | 2 |  | 1 | 1 |  | 5 | 8 |  |  |  |  |  |  |  |  |  |  | 15 | 0.341 |
| KI0010028 | 2000 | Oct | 2 | 1 | 2 |  |  |  | 4 |  |  |  | 1 |  |  |  |  |  |  | 8 | 0.170 |
| KI0010029 | 2000 | Oct | 6 |  | 0 |  |  | 3 | 6 |  |  |  | 1 |  |  |  |  |  |  | 10 | 0.417 |
| KI0010030 | 2000 | Oct | 3 |  |  | 8 |  |  |  |  |  |  |  |  |  |  |  |  |  | 8 | 1.000 |
| KI0010031 | 2000 | Oct | 1 | 372 | 0 | 5 |  |  | 2 | 0 |  |  |  |  |  |  |  | 1 |  | 380 | 0.872 |
| KI0010032 | 2000 | Oct | 2 | 19 | 2 |  |  |  | 2 |  |  |  |  |  |  |  |  |  |  | 23 | 0.535 |
| KI0010033 | 2000 | Oct | 25 |  | 0 |  |  |  | 0 |  |  |  |  |  |  |  |  |  |  | 0 | 0.000 |
| KI0010034 | 2000 | Oct | 6 |  |  |  |  |  | 4 |  |  |  |  |  |  |  |  |  |  | 4 | 0.500 |
| KI0010035 | 2000 | Oct | 25 |  | 0 | 1 |  |  | 4 |  |  |  |  |  |  |  |  |  |  | 5 | 0.455 |
| KI0010036 | 2000 | Oct | 6 |  |  |  |  |  | 1 |  |  |  |  |  |  |  |  |  |  | 1 | 1.000 |
| KI0010037 | 2000 | Oct |  |  |  |  |  |  |  |  |  |  |  |  |  |  |  |  |  |  |  |
| KI0010038 | 2000 | Oct | 6 | 1 | 0 |  |  |  | 6 |  |  |  |  |  |  |  |  |  |  | 7 | 0.467 |
| KI0010039 | 2000 | Oct | 2 |  | 2 |  |  | 3 | 5 |  | 1 |  |  |  |  |  |  |  |  | 11 | 0.306 |
| KI0010040 | 2000 | Oct | 1 | 29 |  |  |  | 4 | 1 |  |  |  |  |  | 1 |  |  |  |  | 35 | 1.000 |
| KI0010041 | 2000 | Oct | 4 |  | 0 | 1 | 47 |  |  |  |  |  |  |  |  |  |  |  |  | 48 | 0.906 |
| KI0010042 | 2000 | Oct | 2 |  | 0 | 7 | 1 |  | 1 |  | 2 |  |  |  |  |  |  |  |  | 11 | 0.407 |
| KI0010043 | 2000 | Oct | 2 |  | 0 | 1 |  |  | 5 |  |  |  |  |  |  |  |  |  |  | 6 | 0.158 |
| KI0010044 | 2000 | Oct | 6 |  |  |  |  |  | 5 |  |  |  |  |  |  |  |  |  |  | 5 | 0.500 |
| KI0010045 | 2000 | Oct | 6 | 4 | 0 |  |  | 2 | 7 |  |  |  | 2 |  |  |  |  |  |  | 15 | 0.682 |
| KI0010046 | 2000 | Oct | 25 |  | 0 |  |  |  | 2 |  | 1 |  |  |  |  |  |  | 1 |  | 5 | 0.714 |
| KI0010047 | 2000 | Oct | 1 | 13 | 0 |  |  |  |  |  |  |  |  |  |  |  |  |  |  | 14 | 0.583 |
| KI0010048 | 2000 | Oct |  |  |  |  |  |  |  |  |  |  |  |  |  |  |  | 1 |  | 1 | 1.000 |
| KI0010049 | 2000 | Oct | 2 | 2 | 6 |  |  |  | 1 |  |  |  | 5 |  |  |  |  | 4 |  | 18 | 0.346 |
| KI0010050 | 2000 | Oct | 25 |  |  |  |  | 1 |  |  |  |  |  |  |  |  |  |  |  | 2 | 1.000 |
| KI0010051 | 2000 | Oct | 6 | 1 | 1 |  | 1 |  | 6 |  |  |  |  |  |  |  |  | 4 |  | 13 | 0.433 |
| KI0010052 | 2000 | Oct | 5 |  | 0 |  |  | 15 | 1 |  |  |  | 2 |  |  |  |  | 19 |  | 37 | 0.804 |
| KI0010053 | 2000 | Oct | 5 | 1 | 1 |  | 11 | 44 | 6 |  |  |  |  |  |  |  |  |  |  | 63 | 0.955 |
| KI0010054 | 2000 | Oct |  |  |  |  |  |  |  |  |  |  |  |  |  |  |  |  |  |  |  |
| KI0010055 | 2000 | Oct | 5 |  |  |  |  | 10 | 1 |  |  |  |  |  |  |  |  |  |  | 12 | 0.857 |
| KI0010056 | 2000 | Oct | 2 |  | 0 |  | 9 | 4 | 1 |  |  |  |  |  |  |  |  |  |  | 14 | 0.350 |
| KI0010057 | 2000 | Oct | 6 |  | 0 |  |  | 11 | 7 |  |  |  |  |  |  |  |  |  |  | 18 | 1.000 |
| KI0010058 | 2000 | Oct |  |  |  |  |  |  |  |  |  |  |  |  |  |  |  |  |  |  |  |
| KI0010059 | 2000 | Oct | 21 |  |  |  |  | 5 |  |  |  |  |  |  |  | 1 |  | 13 |  | 19 | 1.000 |
| KI0010060 | 2000 | Oct | 6 |  | 1 |  |  |  | 9 |  |  |  |  |  |  |  |  |  |  | 10 | 0.625 |
| KI0010061 | 2000 | Oct |  |  |  |  |  |  |  |  |  |  |  |  |  |  |  |  |  |  |  |
| KI0010062 | 2000 | Oct | 6 |  | 4 |  |  |  | 95 |  |  |  |  |  |  |  |  |  |  | 99 | 0.762 |
| KI0010063 | 2000 | Oct | 5 |  | 0 |  |  | 6 | 2 |  |  |  |  |  |  |  |  |  |  | 9 | 0.643 |
| KI0010064 | 2000 | Oct | 2 |  | 0 |  |  |  | 4 |  |  |  |  |  |  |  |  | 7 |  | 11 | 0.367 |
| KI0010065 | 2000 | Oct | 4 |  |  |  | 6 | 5 |  |  |  |  |  |  |  |  |  |  |  | 11 | 1.000 |
| KI0010066 | 2000 | Oct | 6 |  | 4 |  |  |  | 70 |  | 2 |  |  |  |  |  |  |  |  | 76 | 0.974 |
| KI0410001 | 2004 | Oct | 4 |  |  |  | 42 |  |  |  |  |  |  |  |  |  |  |  |  | 42 | 1.000 |
| KI0410002 | 2004 | Oct | 4 |  |  |  | 13 |  |  |  |  |  |  |  |  |  | 1 |  |  | 14 | 1.000 |
| KI0410003 | 2004 | Oct | 4 |  |  |  | 164 |  |  |  |  |  |  |  |  |  |  |  |  | 164 | 0.500 |
| KI0410004 | 2004 | Oct | 4 |  |  |  | 2 |  |  |  |  |  |  |  |  |  |  |  |  | 2 | 1.000 |
| KI0410005 | 2004 | Oct | 4 |  |  |  | 3 |  |  |  |  |  |  |  |  |  |  |  |  | 3 | 1.000 |
| KI0410006 | 2004 | Oct | 4 |  |  |  | 76 |  |  |  |  |  |  |  |  |  |  |  |  | 76 | 1.000 |
| KI0410007 | 2004 | Oct | 17 |  |  |  | 6 |  |  |  |  |  |  |  | 10 |  |  |  |  | 17 | 1.000 |
| KI0410008 | 2004 | Oct | 4 | 5 |  | 84 | 110 |  |  |  |  |  |  |  |  |  |  |  |  | 199 | 1.000 |
| KI0410009 | 2004 | Oct | 4 |  |  |  | 39 |  |  |  |  |  |  |  |  |  |  |  |  | 39 | 1.000 |
| KI0410010 | 2004 | Oct | 4 |  |  | 2 | 27 |  |  |  |  |  |  |  |  |  |  |  |  | 29 | 1.000 |
| KI0410011 | 2004 | Oct | 3 |  |  | 5 | 1 |  |  |  |  |  |  |  |  |  |  |  |  | 6 | 0.857 |
| KI0410012 | 2004 | Oct | 4 |  |  |  | 10 |  |  |  |  |  |  |  |  |  |  |  |  | 10 | 1.000 |
| KI0410013 | 2004 | Oct | 25 |  |  |  | 1 |  |  |  |  |  | 1 |  |  |  |  |  |  | 2 | 0.667 |
| KI0410014 | 2004 | Oct | 4 | 1 |  |  | 91 |  |  |  | 4 |  | 41 |  |  |  |  |  |  | 138 | 0.914 |
| KI0410015 | 2004 | Oct | 4 |  |  | 72 | 104 |  | 1 |  |  |  |  |  |  |  |  |  |  | 178 | 0.249 |
| KI0410016 | 2004 | Oct |  |  |  |  |  |  |  |  |  |  |  |  |  |  |  |  |  |  |  |
| KI0410017 | 2004 | Oct |  |  |  |  |  |  |  |  |  |  |  |  |  |  |  |  |  |  |  |
| KI0410018 | 2004 | Oct | 4 |  |  | 1 | 26 |  |  |  | 1 |  |  |  |  |  |  |  |  | 28 | 1.000 |
| KI0410019 | 2004 | Oct | 4 |  |  | 14 | 17 |  |  |  |  |  |  |  |  |  |  |  |  | 31 | 0.775 |
| KI0410020 | 2004 | Oct | 4 |  |  |  | 92 |  |  |  | 1 |  | 4 |  |  |  |  |  |  | 97 | 1.000 |
| KI0410021 | 2004 | Oct | 3 |  |  | 363 | 164 |  |  |  | 4 |  |  |  |  |  |  |  |  | 531 | 1.000 |
| KI0410022 | 2004 | Oct |  |  |  |  |  |  |  |  |  |  |  |  |  |  |  |  |  |  |  |
| KI0410023 | 2004 | Oct | 4 |  |  |  | 22 |  |  |  | 2 |  | 3 |  |  |  |  |  |  | 27 | 1.000 |
| KI0410024 | 2004 | Oct | 4 |  |  |  | 156 |  |  |  |  |  |  |  |  |  |  |  |  | 156 | 1.000 |
| KI0510001 | 2005 | Oct |  |  |  |  |  |  |  |  |  |  |  |  |  |  | 3 |  |  | 3 | 1.000 |
| KI0510002 | 2005 | Oct | 4 |  |  |  | 40 |  |  |  |  |  |  |  |  |  |  |  |  | 40 | 1.000 |
| KI0510003 | 2005 | Oct | 17 |  |  | 1 |  |  |  |  |  |  |  | 16 | 39 | 74 |  |  |  | 130 | 1.000 |
| KI0510004 | 2005 | Oct | 16 |  |  |  |  |  |  |  |  |  |  | 17 | 13 |  |  |  |  | 30 | 1.000 |
| KI0510005 | 2005 | Oct | 4 |  |  |  | 40 |  |  |  |  |  |  |  | 1 |  | 17 |  |  | 58 | 1.000 |
| KI0510006 | 2005 | Oct | 4 |  |  |  | 45 |  |  |  |  |  |  |  |  |  |  |  |  | 45 | 1.000 |
| KI0510007 | 2005 | Oct | 4 |  |  |  | 51 |  |  |  |  |  |  |  |  |  | 1 |  |  | 52 | 1.000 |
| KI0510008 | 2005 | Oct | 19 |  |  |  | 1 |  |  |  |  |  |  |  | 10 |  | 24 |  |  | 35 | 1.000 |
| KI0510009 | 2005 | Oct | 17 |  |  |  |  |  |  |  |  |  |  |  | 8 |  | 3 |  |  | 11 | 1.000 |
| KI0510010 | 2005 | Oct | 17 |  |  |  |  |  |  |  |  |  |  | 1 | 11 |  | 4 |  |  | 16 | 1.000 |
| KI0510011 | 2005 | Oct | 25 |  |  |  | 4 |  |  |  |  |  |  | 7 | 3 |  | 7 |  |  | 21 | 1.000 |
| KI0510012 | 2005 | Oct | 19 |  |  |  |  |  |  |  |  |  |  |  | 1 |  | 48 |  |  | 49 | 1.000 |
| KI0510013 | 2005 | Oct | 19 |  |  |  | 14 |  |  |  |  |  |  |  | 6 |  | 36 |  |  | 56 | 1.000 |
| KI0510014 | 2005 | Oct | 19 |  |  |  |  |  |  |  |  |  |  |  |  |  | 11 |  |  | 11 | 1.000 |
| KI0510015 | 2005 | Oct | 4 |  | 1 |  | 6 |  | 0 |  |  |  |  |  | 2 |  | 3 |  |  | 12 | 0.923 |
| KI0510016 | 2005 | Oct | 6 |  |  |  |  |  | 15 |  |  |  |  |  |  |  |  |  |  | 15 | 0.750 |
| KI0510017 | 2005 | Oct | 17 |  |  |  |  |  |  |  |  |  |  | 2 | 8 |  |  |  |  | 10 | 1.000 |
| KI0510018 | 2005 | Oct | 17 |  |  |  |  |  | 1 |  |  |  |  |  | 2 |  | 1 |  |  | 4 | 1.000 |
| KI0510019 | 2005 | Oct | 17 |  |  |  | 4 |  |  |  |  |  |  |  | 33 |  | 14 |  |  | 51 | 1.000 |
| KI0510020 | 2005 | Oct | 19 |  |  |  | 3 |  |  |  |  |  |  |  | 23 |  | 38 |  |  | 64 | 1.000 |
| KI0510021 | 2005 | Oct | 19 |  |  |  |  |  |  |  |  |  |  |  |  |  | 4 |  |  | 4 | 1.000 |
| KI0510022 | 2005 | Oct | 4 |  |  |  | 33 |  |  |  |  |  |  |  | 1 |  | 3 |  |  | 38 | 1.000 |
| KI0510023 | 2005 | Oct | 4 |  |  |  | 70 |  | 2 |  |  |  |  |  | 1 |  |  |  |  | 73 | 0.986 |
| KI0510024 | 2005 | Oct | 4 |  |  |  | 155 |  | 10 |  |  |  |  |  | 14 |  |  |  |  | 179 | 0.968 |
| KI0510025 | 2005 | Oct | 4 |  |  |  | 1 |  |  |  |  |  |  |  |  |  |  |  |  | 1 | 1.000 |
| KI0510026 | 2005 | Oct | 17 |  |  |  |  |  |  |  |  |  |  | 3 | 42 |  |  |  |  | 45 | 0.978 |
| KI0510027 | 2005 | Oct | 4 |  |  |  | 65 |  |  | 1 |  |  |  |  |  |  |  |  |  | 66 | 1.000 |
| KI0510028 | 2005 | Oct | 19 |  |  |  | 1 |  |  |  |  |  |  |  | 1 |  | 12 |  |  | 14 | 1.000 |
| KI0510029 | 2005 | Oct | 17 |  | 1 |  | 2 |  |  |  |  |  |  |  | 92 |  | 11 |  |  | 106 | 0.981 |
| KI0510030 | 2005 | Oct | 19 |  |  |  | 6 |  |  |  |  |  |  |  |  |  | 40 |  |  | 46 | 1.000 |
| KI0510031 | 2005 | Oct | 19 |  |  |  |  |  |  |  |  |  |  |  |  |  | 2 |  |  | 2 | 1.000 |
| KI0710001 | 2007 | Oct | 4 |  |  |  | 25 |  | 11 |  |  |  |  |  |  |  |  |  |  | 36 | 0.750 |
| KI0710002 | 2007 | Oct | 1 | 290 |  | 4 | 13 |  | 19 |  |  |  |  |  |  |  |  |  |  | 326 | 0.967 |
| KI0710003 | 2007 | Oct | 4 |  |  |  | 33 |  | 7 | 1 |  |  |  |  |  |  |  |  |  | 41 | 0.707 |
| KI0710004 | 2007 | Oct | 6 |  | 0 |  | 1 |  | 5 |  |  |  |  |  |  |  |  |  |  | 6 | 0.261 |
| KI0710005 | 2007 | Oct | 4 |  |  |  | 25 |  | 6 | 1 |  |  |  |  |  |  |  |  |  | 32 | 0.800 |
| KI0710006 | 2007 | Oct | 3 |  | 1 | 58 | 31 |  | 10 |  |  |  |  |  |  |  |  |  |  | 100 | 0.901 |
| KI0710007 | 2007 | Oct | 6 |  |  |  |  |  | 32 | 1 | 35 |  |  |  |  |  |  |  |  | 68 | 0.840 |
| KI0710008 | 2007 | Oct | 4 |  | 0 |  | 44 |  | 14 |  | 14 |  |  |  |  |  | 2 |  |  | 74 | 0.902 |
| KI0710009 | 2007 | Oct | 4 |  |  |  | 21 |  | 12 |  |  |  |  |  |  |  |  |  |  | 33 | 0.825 |
| KI0710010 | 2007 | Oct | 4 |  |  |  | 46 |  | 0 |  |  |  |  |  |  |  |  |  |  | 46 | 0.979 |
| KI0710011 | 2007 | Oct | 6 | 81 | 1 |  | 31 |  | 62 | 3 | 12 |  |  |  |  |  |  |  |  | 190 | 0.815 |
| KI0710012 | 2007 | Oct | 4 |  |  |  | 134 |  | 8 |  |  |  |  |  |  |  |  |  |  | 142 | 0.973 |
| KI0710013 | 2007 | Oct | 4 |  | 1 | 1 | 117 |  | 0 |  | 2 |  |  |  |  |  |  |  |  | 121 | 0.984 |
| KI0710014 | 2007 | Oct | 4 |  |  |  | 25 |  | 5 | 2 |  |  |  |  |  |  |  |  |  | 32 | 0.744 |
| KI0710015 | 2007 | Oct | 6 |  |  | 1 | 12 |  | 36 | 0 |  |  |  |  |  |  |  |  |  | 50 | 0.347 |
| KI0710016 | 2007 | Oct | 4 |  | 2 |  | 125 |  | 31 | 2 | 1 |  |  |  | 4 |  |  |  |  | 165 | 0.938 |
| KI0710017 | 2007 | Oct | 6 |  | 0 |  |  |  | 37 | 1 | 12 |  |  |  |  |  |  |  |  | 50 | 0.862 |
| KI0710018 | 2007 | Oct | 4 |  | 1 |  | 56 |  | 11 | 1 |  |  |  |  |  |  |  |  |  | 69 | 0.775 |
| KI0710019 | 2007 | Oct | 4 |  |  |  | 18 |  | 2 | 0 |  |  |  |  |  |  |  |  |  | 20 | 0.870 |
| KI0710020 | 2007 | Oct | 6 |  |  |  | 3 |  | 5 |  | 4 |  |  |  |  |  |  |  |  | 12 | 0.706 |
| KI0710021 | 2007 | Oct | 6 |  |  |  | 6 |  | 57 |  | 20 |  |  |  |  |  |  |  |  | 83 | 0.874 |
| KI0710022 | 2007 | Oct | 6 |  | 0 | 4 | 6 |  | 3 |  | 2 |  |  |  |  |  |  |  |  | 15 | 0.682 |
| KI0710023 | 2007 | Oct | 6 |  |  | 5 | 54 | 28 | 64 |  | 1 |  |  |  |  |  |  |  |  | 152 | 0.849 |
| KI0710024 | 2007 | Oct | 6 |  | 0 |  |  |  | 61 |  | 1 |  |  |  |  |  |  |  |  | 62 | 0.689 |
| KI0710025 | 2007 | Oct | 4 |  | 2 | 0 | 101 |  | 7 | 5 |  |  |  |  |  |  |  |  |  | 115 | 0.950 |
| KI0810001 | 2008 | Oct | 4 |  | 0 |  | 5 |  |  |  |  |  |  |  |  |  | 1 |  |  | 6 | 0.857 |
| KI0810002 | 2008 | Oct | 2 |  | 0 |  |  |  |  |  |  |  |  |  |  |  |  |  |  | 0 | 0.000 |
| KI0810003 | 2008 | Oct | 4 |  | 1 | 2 | 21 |  |  |  |  |  |  |  |  |  |  |  |  | 24 | 0.632 |
| KI0810004 | 2008 | Oct |  |  |  |  |  |  |  |  |  |  |  |  |  |  |  |  |  |  |  |
| KI0810005 | 2008 | Oct | 25 |  |  |  | 1 | 1 |  |  |  |  |  |  |  |  |  |  |  | 2 | 1.000 |
| KI0810006 | 2008 | Oct | 25 |  |  |  | 1 | 1 |  |  |  |  |  | 0 |  |  |  |  |  | 2 | 0.667 |
| KI0810007 | 2008 | Oct | 2 |  | 4 |  | 1 |  |  | 1 |  |  |  |  |  |  |  |  |  | 6 | 0.375 |
| KI0810008 | 2008 | Oct | 5 |  | 2 |  |  | 14 |  |  | 1 |  |  |  |  |  |  |  |  | 17 | 0.654 |
| KI0810009 | 2008 | Oct |  |  |  |  |  |  |  |  |  |  |  |  |  |  |  |  |  |  |  |
| KI0810010 | 2008 | Oct | 4 |  | 6 |  | 39 |  |  |  | 1 |  |  |  | 4 |  |  |  |  | 50 | 0.685 |
| KI0810011 | 2008 | Oct | 17 |  | 0 |  |  |  |  |  |  |  |  |  | 45 |  |  |  |  | 45 | 0.900 |
| KI0810012 | 2008 | Oct | 2 |  | 1 | 5 | 4 |  |  |  |  |  |  |  |  |  |  |  | 1 | 11 | 0.647 |
| KI0810013 | 2008 | Oct | 4 |  |  |  | 15 | 2 |  |  | 1 |  |  |  |  |  |  |  |  | 18 | 1.000 |
| KI0810014 | 2008 | Oct | 25 |  |  |  | 2 | 2 |  |  |  |  |  |  |  |  |  |  |  | 4 | 1.000 |
| KI0810015 | 2008 | Oct | 4 |  | 2 |  | 10 | 1 |  |  |  |  |  | 1 |  |  |  |  |  | 14 | 0.824 |
| KI0810016 | 2008 | Oct | 1 | 63 |  |  | 48 |  | 3 |  |  |  |  |  |  |  |  |  |  | 114 | 1.000 |
| KI0810017 | 2008 | Oct | 17 |  | 0 |  | 7 | 20 |  | 1 |  |  |  |  | 60 |  |  |  |  | 88 | 0.978 |
| KI0810018 | 2008 | Oct | 1 | 91 |  |  | 31 | 2 |  |  | 10 |  |  |  | 1 |  |  |  |  | 135 | 0.985 |
| KI0810019 | 2008 | Oct | 2 | 2 | 10 | 2 |  | 12 | 2 |  | 3 |  |  |  | 19 |  |  |  |  | 50 | 0.943 |
| KI0810020 | 2008 | Oct |  |  |  |  |  |  |  |  |  |  |  |  |  |  |  |  |  |  |  |
| KI0810021 | 2008 | Oct | 4 |  | 0 |  | 2 |  |  |  |  |  |  |  | 1 |  |  |  |  | 3 | 0.750 |
| KI0810022 | 2008 | Oct | 4 |  | 2 |  | 27 |  |  |  |  |  |  | 0 |  |  |  |  |  | 29 | 0.879 |
| KI0810023 | 2008 | Oct | 4 | 4 | 3 |  | 29 |  |  |  |  |  |  | 0 | 4 |  |  |  |  | 40 | 0.833 |
| KI0810024 | 2008 | Oct | 4 |  | 1 |  | 54 |  |  |  |  |  |  |  |  |  |  |  |  | 55 | 0.902 |
| KI0810025 | 2008 | Oct | 25 |  | 0 |  |  |  |  |  |  |  |  |  |  |  |  |  | 1 | 1 | 0.500 |
| KI0810026 | 2008 | Oct | 17 | 6 | 4 | 4 | 1 | 14 |  | 1 |  |  |  |  | 52 |  |  |  |  | 83 | 0.965 |
| KI0810027 | 2008 | Oct |  |  |  |  |  |  |  |  |  |  |  |  |  |  |  |  |  |  |  |
| KI0810028 | 2008 | Oct | 17 |  | 1 |  |  |  |  |  |  |  |  | 1 | 15 |  |  |  |  | 17 | 0.944 |
| KI0810029 | 2008 | Oct | 2 |  | 3 |  |  |  |  |  |  |  |  |  | 8 |  | 6 |  |  | 17 | 0.739 |
| KI0810030 | 2008 | Oct | 4 |  | 2 |  | 56 |  |  |  | 1 |  |  |  |  |  |  |  |  | 59 | 1.000 |
| KI0810031 | 2008 | Oct |  |  |  |  |  |  |  |  |  |  |  |  |  |  |  |  |  |  |  |
| KI0810032 | 2008 | Oct | 17 |  | 5 |  |  | 2 |  |  |  |  |  |  | 59 |  |  |  |  | 66 | 0.957 |
| KI0810033 | 2008 | Oct |  |  |  |  |  |  |  |  |  |  |  |  |  |  |  |  |  |  |  |
| KI0810034 | 2008 | Oct | 2 |  | 0 |  |  |  |  |  | 2 |  |  |  | 2 |  |  |  | 1 | 5 | 0.625 |
| KI0810035 | 2008 | Oct | 17 |  |  | 3 |  | 1 |  |  |  |  |  | 0 | 31 |  |  |  |  | 35 | 0.972 |
| KI0810036 | 2008 | Oct | 2 |  | 0 |  | 2 |  |  |  |  |  |  |  |  |  |  |  |  | 2 | 0.250 |
| KI0810037 | 2008 | Oct | 9 |  | 0 |  | 2 |  |  |  | 3 |  |  |  |  |  |  |  |  | 5 | 0.714 |
| KI0810038 | 2008 | Oct | 2 |  | 0 |  |  |  |  |  |  |  |  |  |  |  |  |  |  | 0 | 0.000 |
| KI0810039 | 2008 | Oct | 17 |  | 2 |  | 4 | 35 |  |  | 2 |  |  |  | 96 |  |  |  |  | 139 | 0.979 |
| KI0810040 | 2008 | Oct |  |  |  |  |  |  |  |  |  |  |  |  |  |  |  |  |  |  |  |
| KI0810041 | 2008 | Oct | 17 |  | 0 |  | 7 | 24 |  |  |  |  |  |  | 56 |  |  |  |  | 87 | 0.989 |
| KI0411001 | 2004 | Nov | 13 |  |  |  |  |  |  |  |  | 21 |  |  |  |  |  |  |  | 21 | 1.000 |
| KI0411002 | 2004 | Nov | 3 |  |  | 57 |  |  |  |  |  | 37 |  |  |  |  |  |  |  | 94 | 1.000 |
| KI0411003 | 2004 | Nov | 3 |  |  | 114 | 1 |  |  |  |  |  |  |  |  |  |  |  |  | 115 | 1.000 |
| KI0411004 | 2004 | Nov | 4 |  |  | 9 | 39 |  |  |  |  |  |  |  |  |  |  |  |  | 48 | 0.960 |
| KI0411005 | 2004 | Nov | 4 |  |  | 18 | 28 |  |  |  |  |  |  |  |  |  |  |  |  | 46 | 0.852 |
| KI0411006 | 2004 | Nov | 3 |  |  | 212 |  |  |  |  |  |  |  |  |  |  |  |  |  | 212 | 1.000 |
| KI0411007 | 2004 | Nov | 3 |  |  | 853 | 31 |  |  |  |  |  |  |  |  |  |  |  |  | 885 | 1.000 |
| KI0411008 | 2004 | Nov | 3 |  |  | 4 |  |  |  |  |  |  |  |  |  |  |  |  |  | 4 | 1.000 |
| KI0411009 | 2004 | Nov | 3 |  |  | 20 | 7 |  |  |  |  | 2 |  |  |  |  |  |  |  | 29 | 1.000 |
| KI0411010 | 2004 | Nov | 3 |  |  | 12 |  |  |  |  |  | 1 |  |  |  |  |  |  |  | 20 | 0.870 |
| KI0411011 | 2004 | Nov | 3 |  |  | 124 |  |  |  |  |  | 1 |  |  |  |  |  |  |  | 125 | 1.000 |
| KI0411012 | 2004 | Nov | 3 |  |  | 70 |  |  |  |  |  |  |  |  |  |  |  |  |  | 70 | 0.614 |
| KI0411013 | 2004 | Nov | 3 |  |  | 834 | 4 |  | 1 |  | 1 |  |  |  |  |  |  |  |  | 840 | 1.000 |
| KI0411014 | 2004 | Nov |  |  |  |  |  |  |  |  |  |  |  |  |  |  |  |  |  |  |  |
| KI0411015 | 2004 | Nov | 3 |  |  | 5 |  |  |  |  |  |  | 1 |  |  |  |  |  |  | 6 | 1.000 |
| KI0411016 | 2004 | Nov |  |  |  |  |  |  |  |  |  |  |  |  |  |  |  |  |  |  |  |
| KI0411017 | 2004 | Nov | 3 |  |  | 66 |  |  |  |  |  |  |  |  |  |  |  |  |  | 66 | 0.917 |
| KI0411018 | 2004 | Nov | 3 |  |  | 34 | 22 |  |  |  |  |  |  |  |  |  |  |  |  | 56 | 0.875 |
| KI0411019 | 2004 | Nov | 3 | 1 |  | 132 | 48 |  |  |  |  |  |  |  |  |  |  |  |  | 181 | 0.342 |
| KI0411020 | 2004 | Nov |  |  |  |  |  |  |  |  |  |  |  |  |  |  |  |  |  |  |  |
| KI0411021 | 2004 | Nov | 13 |  |  | 1 |  |  |  |  |  | 30 | 3 |  |  |  |  |  |  | 34 | 1.000 |
| KI0411022 | 2004 | Nov | 3 |  |  | 645 | 1 |  | 1 |  |  |  |  |  |  |  |  |  |  | 647 | 1.000 |
| KI0411023 | 2004 | Nov | 3 |  |  | 28 | 5 |  |  |  |  | 1 |  |  |  |  |  |  |  | 35 | 0.745 |
| KI0411024 | 2004 | Nov | 3 |  |  | 18 |  |  |  |  |  |  | 3 |  |  |  |  |  |  | 21 | 0.913 |
| KI0411025 | 2004 | Nov | 3 |  |  | 11 |  |  |  |  |  |  |  |  |  |  |  |  |  | 11 | 0.688 |
| KI0411026 | 2004 | Nov | 3 |  |  | 60 | 6 |  |  |  |  |  |  |  |  |  |  |  |  | 71 | 0.922 |
| KI0411027 | 2004 | Nov |  |  |  |  |  |  |  |  |  |  |  |  |  |  |  |  |  |  |  |
| KI0411028 | 2004 | Nov | 13 |  |  | 10 |  |  |  |  |  | 21 |  |  |  |  |  |  |  | 36 | 0.857 |
| KI0411029 | 2004 | Nov | 3 |  |  | 37 |  |  |  |  |  | 2 | 5 |  |  |  |  |  |  | 45 | 0.818 |
| KI0411030 | 2004 | Nov |  |  |  |  |  |  |  |  |  |  |  |  |  |  |  |  |  |  |  |
| KI0411031 | 2004 | Nov | 3 |  |  | 9 |  |  |  |  |  | 5 | 3 |  |  |  |  |  |  | 17 | 0.708 |
| KI0411032 | 2004 | Nov | 3 |  |  | 13 |  |  |  |  |  | 2 | 1 |  |  |  |  |  |  | 16 | 0.615 |
| KI0411033 | 2004 | Nov | 3 |  |  | 22 |  |  |  |  |  |  | 7 |  |  |  |  |  | 232 | 264 | 0.971 |
| KI0411034 | 2004 | Nov | 3 |  |  | 110 |  |  |  |  |  | 1 | 4 |  |  |  |  |  |  | 115 | 1.000 |
| KI0411035 | 2004 | Nov |  |  |  |  |  |  |  |  |  |  |  |  |  |  |  |  |  |  |  |
| KI0411036 | 2004 | Nov | 3 |  |  | 21 | 0 |  |  |  |  | 14 | 10 |  |  |  |  |  |  | 45 | 0.789 |
| KI0411037 | 2004 | Nov |  |  |  |  |  |  |  |  |  |  |  |  |  |  |  |  |  |  |  |
| KI0411038 | 2004 | Nov | 13 |  |  | 2 |  |  |  |  | 2 | 20 | 1 |  |  |  |  |  |  | 27 | 1.000 |
| KI0411039 | 2004 | Nov |  |  |  |  |  |  |  |  |  |  |  |  |  |  |  |  |  |  |  |
| KI0411040 | 2004 | Nov | 4 |  |  | 19 | 29 |  |  |  |  | 2 |  |  |  |  |  |  |  | 50 | 1.000 |
| KI0411041 | 2004 | Nov | 3 |  |  | 24 |  |  |  |  |  |  |  |  |  |  |  |  |  | 38 | 0.864 |
| KI0411042 | 2004 | Nov | 3 |  |  | 7 |  |  |  |  |  | 1 | 1 |  |  |  |  |  |  | 10 | 0.833 |
| KI0411043 | 2004 | Nov | 14 |  |  | 24 |  |  |  |  |  |  | 25 |  |  |  |  |  |  | 50 | 1.000 |
| KI0411044 | 2004 | Nov |  |  |  |  |  |  |  |  |  |  |  |  |  |  |  |  |  |  |  |
| KI0411045 | 2004 | Nov | 3 |  |  | 11 | 13 | 1 |  |  |  |  |  |  |  |  |  |  |  | 25 | 0.862 |
| KI0511001 | 2005 | Nov | 1 | 370 | 0 | 10 | 153 |  |  |  |  |  |  |  |  |  |  |  |  | 533 | 0.994 |
| KI0511002 | 2005 | Nov | 4 |  |  |  | 62 |  |  |  |  |  |  |  |  |  |  |  |  | 62 | 1.000 |
| KI0511003 | 2005 | Nov | 4 |  |  |  | 10 |  |  |  |  |  |  |  |  |  |  |  |  | 10 | 1.000 |
| KI0511004 | 2005 | Nov | 4 |  |  |  | 8 |  |  |  |  |  |  |  |  |  |  |  |  | 8 | 1.000 |
| KI0511005 | 2005 | Nov | 4 |  |  |  | 93 |  |  |  |  |  |  |  |  |  |  |  |  | 93 | 1.000 |
| KI0511006 | 2005 | Nov | 4 |  |  | 3 | 51 |  |  |  |  |  |  |  |  |  |  |  |  | 54 | 1.000 |
| KI0511007 | 2005 | Nov | 4 |  |  |  | 102 |  |  |  |  |  |  |  |  |  |  |  |  | 102 | 1.000 |
| KI0511008 | 2005 | Nov | 4 |  |  |  | 139 |  |  |  |  |  |  |  |  |  |  |  |  | 139 | 1.000 |
| KI0511009 | 2005 | Nov |  |  |  | 1 | 1 |  |  |  |  |  |  |  |  |  |  |  |  | 2 | 1.000 |
| KI0511010 | 2005 | Nov | 4 |  |  |  | 23 | 8 |  |  |  |  |  |  |  |  |  |  |  | 31 | 1.000 |
| KI0511011 | 2005 | Nov | 4 |  |  |  | 6 |  | 1 |  |  |  |  |  | 2 |  |  |  |  | 10 | 1.000 |
| KI0511012 | 2005 | Nov | 4 |  |  | 164 | 433 |  |  |  |  |  |  |  |  |  |  |  |  | 597 | 1.000 |
| KI0511013 | 2005 | Nov | 4 |  | 2 | 29 | 251 |  |  |  |  |  |  |  |  |  |  |  |  | 282 | 0.993 |
| KI0511014 | 2005 | Nov | 4 |  |  |  | 34 |  | 6 |  |  |  |  |  |  |  |  |  |  | 40 | 1.000 |
| KI0511015 | 2005 | Nov | 4 |  |  |  | 5 |  |  |  |  |  |  |  |  |  |  |  |  | 5 | 1.000 |
| KI0711001 | 2007 | Nov | 4 |  |  | 1 | 258 |  | 1 |  |  |  |  |  |  |  |  |  |  | 260 | 0.996 |
| KI0711002 | 2007 | Nov | 4 |  |  | 32 | 515 |  | 2 | 2 |  |  |  |  |  |  |  |  |  | 551 | 0.998 |
| KI0711003 | 2007 | Nov |  |  |  |  |  |  |  |  |  |  |  |  |  |  |  |  |  |  |  |
| KI0711004 | 2007 | Nov | 4 | 2 |  |  | 78 |  |  |  |  |  |  |  |  |  |  |  |  | 80 | 1.000 |
| KI0711005 | 2007 | Nov | 4 | 10 |  | 6 | 65 |  |  |  |  |  |  |  |  |  |  |  |  | 81 | 1.000 |
| KI0711006 | 2007 | Nov | 3 |  |  | 138 | 91 |  |  |  | 1 |  |  |  |  |  |  |  |  | 230 | 1.000 |
| KI0711007 | 2007 | Nov | 4 |  |  | 20 | 68 | 1 |  |  |  |  |  |  |  |  |  |  |  | 89 | 0.947 |
| KI0711008 | 2007 | Nov | 4 | 104 |  | 62 | 445 |  |  | 0 |  |  |  |  |  |  |  |  |  | 611 | 1.000 |
| KI0711009 | 2007 | Nov |  |  |  |  |  |  |  |  |  |  |  |  |  |  |  |  |  |  |  |
| KI0711010 | 2007 | Nov | 3 |  |  | 155 | 103 |  |  | 1 |  |  |  |  |  |  |  |  |  | 261 | 1.000 |
| KI0711011 | 2007 | Nov |  |  |  |  |  |  |  |  |  |  |  |  |  |  |  |  |  |  |  |
| KI0711012 | 2007 | Nov | 3 |  |  | 108 | 60 |  |  | 1 | 2 |  |  |  |  |  |  |  |  | 171 | 1.000 |
| KI0711013 | 2007 | Nov | 3 | 1 |  | 427 | 316 |  |  |  |  |  |  |  |  |  |  |  |  | 745 | 0.999 |
| KI0711014 | 2007 | Nov |  |  |  |  |  |  |  |  |  |  |  |  |  |  |  |  |  |  |  |
| KI0711015 | 2007 | Nov |  |  |  |  |  |  |  |  |  |  |  |  |  |  |  |  |  |  |  |
| KI0711016 | 2007 | Nov |  |  |  |  |  |  |  |  |  |  |  |  |  |  |  |  |  |  |  |
| KI0711017 | 2007 | Nov |  |  |  |  |  |  |  |  |  |  |  |  |  |  |  |  |  |  |  |
| KI0711018 | 2007 | Nov |  |  |  |  |  |  |  |  |  |  |  |  |  |  |  |  |  |  |  |
| KI0711019 | 2007 | Nov | 4 |  |  | 21 | 95 |  |  |  |  |  |  |  |  |  |  |  |  | 116 | 0.967 |
| KI0711020 | 2007 | Nov | 4 |  |  | 2 | 297 |  |  |  | 1 |  |  |  |  |  |  |  |  | 300 | 0.997 |
| KI0711021 | 2007 | Nov |  |  |  |  |  |  |  |  |  |  |  |  |  |  |  |  |  |  |  |
| KI0711022 | 2007 | Nov | 4 |  |  | 1 | 357 |  |  |  |  |  |  |  |  |  |  |  |  | 358 | 1.000 |
| KI0711023 | 2007 | Nov |  |  |  |  |  |  |  |  |  |  |  |  |  |  |  |  |  |  |  |
| KI0811001 | 2008 | Nov | 4 |  |  | 1 | 68 |  |  |  |  |  |  |  |  |  |  |  |  | 69 | 1.000 |
| KI0811002 | 2008 | Nov | 4 |  | 0 | 4 | 41 |  |  |  |  |  |  |  |  |  |  |  |  | 45 | 0.978 |
| KI0811003 | 2008 | Nov | 3 |  | 4 | 82 | 6 | 18 |  |  | 1 |  |  |  |  |  |  |  |  | 111 | 0.841 |
| KI0811004 | 2008 | Nov | 1 | 86 | 14 |  | 58 |  |  |  |  |  |  |  |  |  |  |  |  | 165 | 0.859 |
| KI0811005 | 2008 | Nov | 3 | 28 | 0 | 184 | 102 | 15 |  | 1 |  |  |  |  |  |  |  |  |  | 341 | 0.974 |
| KI0811006 | 2008 | Nov | 3 | 2 | 1 | 126 | 24 | 31 |  | 1 |  |  |  |  |  |  |  |  |  | 185 | 0.969 |
| KI0811007 | 2008 | Nov | 5 |  |  | 1 | 18 | 27 |  |  |  |  |  |  |  |  |  |  |  | 46 | 1.000 |
| KI0811008 | 2008 | Nov | 3 |  |  | 100 | 34 | 4 |  | 1 |  |  |  |  |  |  |  |  |  | 142 | 0.570 |
| KI0811009 | 2008 | Nov | 3 |  | 1 | 98 | 10 | 24 |  |  |  |  |  |  |  |  |  |  |  | 133 | 1.000 |
| KI0811010 | 2008 | Nov | 4 | 3 | 3 |  | 70 | 25 |  |  |  |  |  |  |  |  |  |  |  | 112 | 0.903 |
| KI0811011 | 2008 | Nov | 3 | 3 | 4 | 466 | 24 | 13 |  |  |  |  |  |  |  |  |  |  |  | 523 | 0.981 |
| KI0811012 | 2008 | Nov | 4 |  | 8 | 16 | 56 | 22 |  |  |  |  |  |  |  |  |  |  |  | 111 | 0.982 |
| KI0811013 | 2008 | Nov | 3 |  |  | 80 |  | 19 |  |  |  |  |  |  |  |  |  |  |  | 99 | 1.000 |
| KI0811014 | 2008 | Nov | 4 |  |  | 2 | 8 |  |  |  |  |  |  |  |  |  |  |  |  | 10 | 1.000 |
| KI0811015 | 2008 | Nov | 3 |  | 8 | 243 | 6 |  |  |  |  |  |  |  |  |  |  |  |  | 257 | 1.000 |
| KI0811016 | 2008 | Nov | 4 |  |  |  | 53 | 19 |  | 0 |  |  |  |  |  |  |  |  |  | 72 | 0.960 |
| KI0811017 | 2008 | Nov | 4 |  | 0 |  | 7 |  |  |  |  |  |  |  |  |  |  |  |  | 7 | 0.778 |
| KI0811018 | 2008 | Nov | 3 |  |  | 309 | 146 |  |  | 1 |  |  |  |  |  |  |  |  |  | 457 | 0.977 |
| KI0811019 | 2008 | Nov | 3 |  |  | 183 | 69 |  |  |  |  |  |  |  |  |  |  |  |  | 252 | 0.985 |
| KI0811020 | 2008 | Nov | 4 |  |  | 14 | 374 |  |  |  |  |  |  |  |  |  |  |  |  | 390 | 1.000 |
| KI0811021 | 2008 | Nov | 3 |  |  | 310 | 68 | 1 |  |  |  |  |  |  |  |  |  |  |  | 380 | 0.975 |
| KI0811022 | 2008 | Nov | 4 |  |  | 64 | 294 |  |  |  |  |  |  |  |  |  |  |  |  | 358 | 1.000 |
| KI0811023 | 2008 | Nov | 4 |  | 0 | 14 | 252 |  |  |  |  |  |  |  |  |  |  |  |  | 266 | 0.996 |
| KI0811024 | 2008 | Nov | 4 |  | 0 | 1 | 164 |  |  | 2 |  |  |  |  |  |  |  |  |  | 168 | 0.988 |
| KI0811025 | 2008 | Nov | 3 |  | 1 | 6 | 3 |  |  |  |  |  |  |  |  |  |  |  |  | 12 | 0.047 |
| KI0811026 | 2008 | Nov | 4 |  |  | 4 | 191 |  |  |  |  |  |  |  |  |  |  |  |  | 195 | 1.000 |
| KI0811027 | 2008 | Nov | 4 |  |  | 54 | 218 |  |  |  |  |  |  |  |  |  |  |  |  | 275 | 0.989 |
| KI0811028 | 2008 | Nov | 4 |  |  | 2 | 45 |  |  |  |  |  |  |  |  |  |  |  |  | 48 | 1.000 |
| KI0811029 | 2008 | Nov | 4 |  |  | 100 | 497 |  |  |  |  |  |  |  | 6 |  |  |  |  | 603 | 0.921 |
| KI0811030 | 2008 | Nov | 4 | 1 |  | 55 | 201 |  |  |  |  |  |  |  |  |  |  |  |  | 257 | 0.992 |
| KI0811031 | 2008 | Nov | 4 |  |  | 25 | 281 | 2 |  |  |  |  |  |  |  |  |  |  |  | 309 | 1.000 |
| KI0811032 | 2008 | Nov | 4 |  | 0 |  | 88 | 19 |  |  |  |  |  |  |  |  |  |  |  | 108 | 0.991 |
| KI0811033 | 2008 | Nov | 4 |  |  | 8 | 151 |  |  |  |  |  |  |  |  |  |  |  |  | 159 | 1.000 |
| KI0811034 | 2008 | Nov | 4 |  |  | 3 | 237 |  |  |  |  |  |  |  |  |  |  |  |  | 240 | 0.996 |
| KI0811035 | 2008 | Nov | 3 |  |  | 87 | 79 |  |  |  |  |  |  |  |  |  |  |  |  | 166 | 1.000 |
| KI0811036 | 2008 | Nov | 4 |  |  | 49 | 148 | 16 |  |  |  |  |  |  |  |  |  |  |  | 213 | 0.995 |
| KI0811037 | 2008 | Nov | 4 |  |  | 2 | 20 | 5 |  |  |  |  |  |  |  |  |  |  |  | 27 | 0.964 |
| KI0811038 | 2008 | Nov | 4 |  |  | 61 | 133 |  |  |  |  |  |  |  |  |  |  |  |  | 194 | 0.985 |
| KI0811039 | 2008 | Nov | 4 |  |  | 88 | 204 |  |  |  |  |  |  |  |  |  |  |  |  | 292 | 0.993 |

Species: 1 = *Tubocapsicum anomalum*, 2 = *Swida macrophylla*, 3 = *Rosa multiflora*, 4 = *Vibrnum dilatatum*, 5 = *Malus tschonoskii*, 6 = *Pourthiaea villosa*, 7 = *Zanthoxylum piperitum*, 9: *Perilla frutescens*, 13: *Viscum album*, 14: *Amphicarpaea edgeworthii*, 16: *Cornus kousa*, 17: *Vitis flexuosa*, 18: *Berchemia racemosa*, 19: *Schisandra nigra*, 21: *Sorbus japonica*, X: unidentified. *DPS*: dominant plant species (if there was no dominant species, we recorded as "25").
